# Personalized Smartphone-Enabled Assessment of Blood Pressure and Its Treatment During the SARS-CoV-2 COVID-19 Pandemic in Patients From the CURE-19 Study: Longitudinal Observational Study

**DOI:** 10.2196/53430

**Published:** 2024-12-03

**Authors:** Leanne Richardson, Nihal Noori, Jack Fantham, Gregor Timlin, James Siddle, Thomas Godec, Mike Taylor, Charles Baum

**Affiliations:** 1 Closed Loop Medicine Cambridge United Kingdom; 2 Encore Health Chicago, IL United States

**Keywords:** digital diary, hypertension, blood pressure, remote monitoring, smartphone app, mobile phone, app, monitoring, COVID-19, SARS-CoV-2, digital intervention, management, observational study, deployment, feasibility, use, safety, medication, symptoms, community, systolic, diastolic, utilization

## Abstract

**Background:**

The use of digital interventions by patients for remote monitoring and management of health and disease is increasing. This observational study examined the feasibility, use, and safety of a digital smartphone app for routine monitoring of blood pressure (BP), medication, and symptoms of COVID-19 during the COVID-19 pandemic.

**Objective:**

The objective of this study was to deploy and test electronic data recording using a smartphone app developed for routine monitoring of BP in patients with primary hypertension. We tested the app for ease of data entry in BP management and tracking symptoms of new-onset COVID-19 to determine if participants found this app approach useful and sustainable.

**Methods:**

This remote, decentralized, 12-week, prospective, observational study was conducted in a community setting within the United States. Participants were approached and recruited from affiliated sites where they were enrolled in an ongoing remote decentralized study (CURE-19) of participants experiencing the COVID-19 pandemic. Potential participants were asked to complete a digital screener to determine eligibility and given informed consent forms to read and consent to using the Curebase digital platform. Following enrollment, participants downloaded the digital app to their smartphones for all data collection. Participants recorded daily BP, associated medication use, and emergent symptoms associated with SARS-CoV-2 infection. In addition, usability (adherence, acceptability, and user experience) was assessed using standard survey questions. Adverse events were collected based on participant self-report. Compliance and engagement were determined from user data entry rates. Feasibility and participant feedback were assessed upon study completion using the User Experience Questionnaire.

**Results:**

Of the 389 participants who enrolled in and completed the study, 380 (98%) participants downloaded and entered BP routines in week 1. App engagement remained high; 239 (62.9%) of the 380 participants remained in the study for the full 12-week observation period, and 201 (84.1%) of the 239 participants entered full BP routines into the digital app 80% or more of the time. The smartphone app scored an overall positive evaluation as assessed by the User Experience Questionnaire and was benchmarked as “excellent” for domains of perspicuity, efficiency, and dependability and “above average” for domains of attractiveness and stimulation. Highly adherent participants with hypertension demonstrated well-controlled BP, with no significant changes in average systolic or diastolic BP between week 1 and week 12 (all *P*>.05). Participants were able to record BP medications and symptoms of SARS-CoV-2 infection. No adverse events attributable to the use of the smartphone app were reported during the observational period.

**Conclusions:**

The high retention, engagement and acceptability and positive feedback in this study demonstrates that routine monitoring of BP and medications using a smartphone app is feasible for patients with hypertension in a community setting. Remote monitoring of BP and data collection could be coupled with hypertensive medication in a combination product (drug+digital) for precision management of hypertension.

## Introduction

Hypertension is the leading preventable cause of premature death worldwide [1]. Prevalence of hypertension in the United States is 44% to 49% with an estimated 116 million having the disease [2]. Physician inertia (inadequate up-titration of treatment, especially from monotherapy) and poor patient adherence to treatment (especially when based on multiple pills) are now recognized as major factors contributing to poor blood pressure (BP) control [1,3,4].

During the COVID-19 pandemic, social distancing was a priority measure to limit infection in the general population. This measure alongside the redeployment of clinicians to frontline medicine restricted the medical management of hypertension. In turn, this led to the desire to move as much medical management as possible to arms-length support. To meet this need, health care providers rapidly moved to remote monitoring and patient engagement tools where possible. This trend gained momentum, and as a result, over the past 3 years, a 38-fold increase in the use of remote communication technology to deliver health care services (telehealth) in the United States has been observed [5]. This, alongside a strong patient preference to use telehealth services for ordering medications, clinical visit preparation, and receiving educational material and test results, suggests that widespread adoption of telehealth services by health care providers, insurance companies, and patients will continue [6]. Digitization and automation of these services not only can increase patient access to health services but can also build efficiencies within the health care system.

These recent changes have occurred against a backdrop of longer-term technical developments, allowing the delivery of personalized drug treatment to an individual to get much closer to being feasible. This; the use of electronic diaries; and eventually, as more data are collected over time, decision support by mobile apps, could make that a reality. Our team (Closed Loop Medicine Ltd [CLM]) has expertise in the design and development of electronic apps to develop patient treatment solutions that allow combinations of drug and nondrug therapies. One such solution is a smartphone app created by CLM and coproduced by patients through patient engagement and user testing. This prototype app allows patients’ BP diary entries, collection of hypertensive adverse drug reactions, and other patient-reported data to be routed to a study database for analysis and surveillance.

When the full scale of the COVID-19 pandemic became apparent, we responded by adapting the prototype app to collect COVID-19 symptoms and hypertension drug regimen information, using the existing patient experience and data collection technology. This corresponded to our early design thinking for a general-purpose platform. The app was made available to study participants through Google and Apple app stores. This allowed deployment of the app into the community setting within the United States in an observational study facilitated by our study partner Curebase. Additional data collection and integration components and a financial reconciliation program were developed to facilitate the study’s operational aspects.

The information gained from this study regarding BP measurements, type of drug taken and symptom data, and the learnings and experience of delivering this study will inform future product development plans and future iterations of the hypertension platform to best meet the needs of health care providers and patients. Furthermore, the information gained will provide evidence that digitization of health care is feasible and a valid means of remote management of hypertension within the existing health care system.

Therefore, the observational study aimed to rapidly modify, deploy, and test a digital smartphone app for routine monitoring of BP, BP-associated medications, and symptoms of SARS-CoV-2 infection in the community setting during the COVID-19 pandemic. This testing would provide valuable information for further development of the app before use in future interventional clinical trials. Alongside this, we examined core interactions and use of the app by patients with hypertension, assessed by participant engagement, compliance, and a User Experience Questionnaire (UEQ) [7]. From a clinical point of view, we assessed BP control in a community setting within the United States and whether BP drug use or level of BP control affected the risk of SAR-CoV-2 infection. We also assessed the safety of participants using the app. In addition, this study established a well-defined cohort of patients ready for recruitment into an interventional trial, should we, or other research groups, need this on account of new insights into COVID-19 and the requirement for rapid study execution.

## Methods

### Personalized Electronic Record Development and Deployment

A mobile app was created for use in this observational study setting. The app was developed as a data collection interface and modified to additionally collect COVID-19 symptoms as well as current antihypertensive medications, based on a curated set of hypertension drugs and doses that were identified as potentially impacting COVID-19 symptoms. These modifications, along with the existing prototype app experience and an incentives scheme rewarding patient adherence, were used to promote patient engagement in the data collection process. Table 1 lists the standard behavior change techniques [8] and the corresponding participant engagement features used to encourage data entry in the study. Some of the corresponding user interfaces used in the study are shown in Multimedia Appendix 1.

As shown in the system diagram (Multimedia Appendix 2), the mobile app was supported by several shared backend services hosted in Amazon Web Services. These were extended to support storage of the new data and to allow the data to be shared with CLM’s study partner Curebase. A financial reconciliation service was used, where payment eligibility was calculated on the basis of study participation level, then passed on to Curebase in support of a regularly scheduled payment process.

New Google and Apple app store entries were created and configured so that patients in the United States were able to install and run the app. Patient study enrollment was managed using a previously established approach based on a unique patient identifier and an enrollment code shared with the patient, which was then linked to their mobile device. A new set of patient identifiers and enrollment codes was generated and shared with Curebase, which integrated these into their patient training and onboarding process.

**Table 1:** Participant engagement features.

Behavior changes technique taxonomy [8]	Participant engagement features
Self-monitoring of behavior: Establish a method for the person to monitor and record their behaviors as part of a behavior change strategy.	Blood pressure monitoring: Enable participants to enter their blood pressure readings into the app. COVID-19 symptom monitoring: Enable participants to enter any COVID-19 symptoms they experience. Hypertension medication monitoring: Enable participants to maintain a list of their current hypertensive medications.
Demonstration of the behavior: Provide an observable sample of the performance of the behavior, directly in person or indirectly, for example, through film, pictures, for the person to aspire to or imitate.	Blood pressure measurement demonstration: Educate participants on the correct way to take consistent blood pressure readings.
Instruction on how to perform a behavior: Advise or agree on how to perform the behavior.	COVID-19 symptom information: Provide participants with clear information about COVID-19 symptoms so they can correctly identify any symptoms they are experiencing.
Habit formation: Prompt rehearsal and repetition of the behavior in the same context repeatedly so that the context elicits the behavior.	Morning and evening routine management: Enable consistent routine formation by providing a morning and evening data entry ritual.
Prompts or cues: Introduce or define environmental or social stimulus with the purpose of prompting or cueing the behavior. The prompt or cue would normally occur at the time or place of performance.	Smartphone reminders: Enable participants to set routine reminders to enter data into the app.
Feedback on outcome of behavior: Monitor and provide feedback on the outcome of performance of the behavior.	Data history: Provide participants with clear diagrams of previous blood pressure and COVID-19 symptom entries.
Incentive (outcome): Inform that a reward will be delivered if and only if there has been effort or progress in achieving the behavioral outcome.	Participant incentives: Participants who downloaded the app successfully were given US $10 and then an additional US $10 each week for 12 weeks if all study activities were completed. Participants could continue using the app after the 12 weeks but were not compensated for completion of activities.

### Study Design

This remote, decentralized, 12-week, prospective, observational study was conducted in the community with no participant visits to investigational sites during the COVID-19 pandemic. The study consisted of an observational period of 12 weeks, with an optional 12-week extension. Participants recorded daily BP (morning and evening) and associated medication use, as well as any emergent symptoms indicative of SARS-CoV-2 infection.

### Participants

Participants were aged 18 years and older with hypertension, defined as receiving prescription drug treatment (minimum of 1 drug) to reduce BP. To take part, participants had to be willing to provide informed consent; be able to speak, read, and comprehend English; and possess a suitable home BP monitoring device (as a BP monitor would not be provided to participants) and a suitable smartphone (to support iOS versions 10.0 [Apple Inc] and newer or Android versions 5.0 [Google] and newer; minimum storage space required to install the digital app) that they could independently use. Main exclusions included any known or suspected COVID-19 symptoms at enrollment, comorbidities incompatible with study participation (ie, that would inhibit completion of daily smartphone app entries), and limited or no understanding of spoken or written English.

Participants were recruited to the study through internet-based recruitment to a larger study called CURE-19 being undertaken by Curebase (contract research organization [CRO]). Study personnel at clinical sites affiliated with the CRO approached and recruited participants from their patient population. Those who were interested in taking part of the study were then screened for eligibility, and if deemed eligible, they continued to the enrollment phase. Digital recruitment methods including recruitment campaigns through social media and internet advertisement were also used. Potential participants who encountered recruitment information through social media or on the internet expressed interest by clicking on the content, which led them to a study landing page website. To assess eligibility, a digital screener containing relevant questions was used, asking potential participants questions to determine their eligibility. All participants who submitted screeners were asked to provide their email address in order to create an account. Screening responses were automatically analyzed and an outcome for the patient was generated, either eligible or ineligible. If eligible, patients were given informed consent forms to read and were consented using the CRO’s digital platform, either remotely on their own personal device or in the presence of study personnel. Electronic signatures were captured and stored electronically in the Curebase platform.

### Study Procedures

Following consent, participants were given instructions through the CRO’s remote onboarding system to download the digital smartphone app from the app store on their personal smartphone and complete the baseline data entry in the app. In some instances, remote onboarding also included telephone calls as well as emails sent to the participants. Participants were then asked to enter routine daily recordings of BP (morning and evening), BP medication use, and emergent symptoms of SAR-CoV-2 infection for 12 weeks. Following the initial 12-week observational period, participants could continue to an additional optional 12-week extension period. Demographic and adverse event information was collected from participants by self-report and electronic medical records by an electronic case report form. Participants received internet-based surveys to complete in order to collect self-reported data. At the completion of 12 weeks or 24 weeks, the UEQ was sent to the participants as a Google Form to allow participants to give their evaluation of the smartphone app they used during the study.

### Outcomes

The main objective of this study was to deploy and test a personalized electronic record in the form of a digital smartphone app to participants with primary hypertension for routine monitoring of BP in a community setting within the United States. We used this app to assess daily BP control within this population. We also assessed interactions with the app over the 12-week observational period, including compliance to BP monitoring routines (percentage of patients entering a BP diary entry on 80% or more occasions) and patient feedback and satisfaction using the smartphone app as assessed by the UEQ. Other outcomes of interest included assessment of antihypertensive medication use and emergent symptoms of SARS-CoV-2 infection including cough, fever, and shortness of breath. The safety of participants in relation to the use of the app was assessed through self-reporting of adverse events (AEs) and serious adverse events (SAEs). Exploratory outcomes included analysis of associations of BP antihypertensive drug use as well as BP level with the incidence of SARS-CoV-2 infection and the severity of COVID-19 outcomes, to determine if antihypertensive drug use or the level of BP control can affect severity of COVID-19 illness or pneumonia.

### Statistical Analysis

This study’s exploratory nature made finding a specific sample size impractical. Given this study’s low risk, there was no cap on enrollment and the intent was to enroll as many participants as possible within the recruitment time period. As this was an observational study, analyses included basic descriptive statistics and statistical tests to assess differences between groups. Analysis groups were defined based on compliance with BP data capture. We planned to analyze patient outcomes, but due to the lack of disease outcomes observed in the study, this was not done. Adherence to BP monitoring routines was assessed by calculating the percentage of patients completing a full BP diary routine entry on 80% or more occasions (a full routine consisted of 3 BP measurements and routines were twice daily—morning and evening). Complete adherence to the digital diary was defined in terms of full routines consisting of morning BP in triplicate with medications summary, or an evening BP triplicate along with a symptoms report.

### Ethical Considerations

This study was reviewed and approved by IntegReview Institutional Review Board (later became Advarra), which provided a favorable opinion to conduct this study in the United States. Participants who were deemed eligible and after submitting their screeners and signed informed consent forms using electronic signature had their forms stored in the Curebase platform. Participants were able to withdraw from the study at any time. They were able to withdraw consent as well as have all archived clinical data discarded. All information was confidential and data were pseudonymized. Institutional review board–approved small pro rata payments were offered to participants as compensation for participation based on the amount of time in the study and the number of data entry routines completed (US $10 for successfully downloading the app and an additional US $10 for each week of participation up to 12 weeks total).

## Results

### Overview of Study, Procedures, and Patient Population

**Figure 1:**
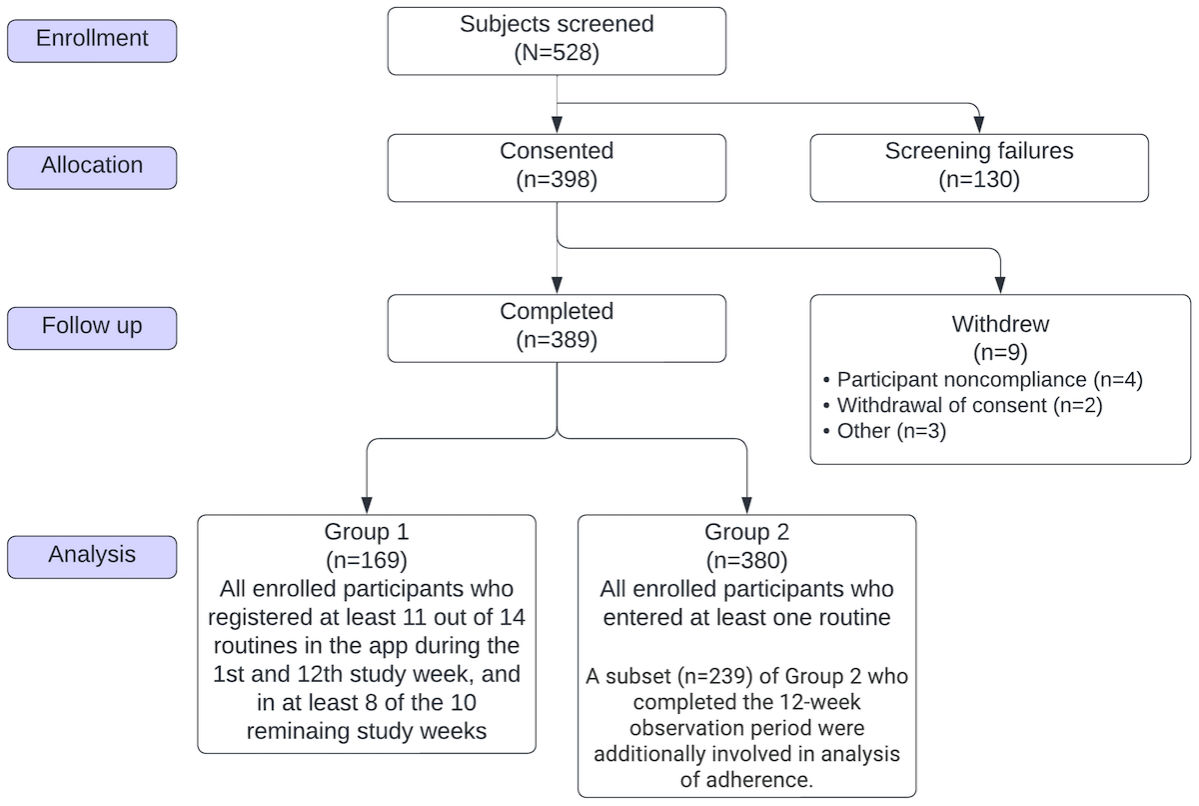
Details of enrollment into the trial.

A total of 528 participants were screened for entry into the study (Figure 1). From August 2020 to November 2020, a total of 398 patients with hypertension were enrolled, with 389 (98%) participants completing the study. The last participant completed follow-up in February 2021, with 9 participants being lost to follow up. A summary of participant disposition is shown in Figure 1. Participants were analyzed in 2 main groups, with group 1 consisting of 169 participants who were highly compliant at data entry into the smartphone app (participants who registered at least 11 of 14 routines in the app during the 1st and 12th study week, and in at least 8 of the 10 remaining study weeks). At least 80% of routine entries was considered to be the benchmark in this study based on Haynes and colleagues’ [9] definition of sufficient adherence of individuals taking antihypertensive medication being ≥80%.

The other main group, group 2, consisted of 380 participants (overall study population), which included all participants who had entered 1 or more routines into the app. There were 9 participants who did not enter any routines. A subset of group 2 was defined for the purpose of adherence consisting of 239 (62.9%) participants who remained in the study until the end of the 12-week observation period.

A summary of demographic data of all participants is presented in Table 2. The average age of the population was 58.1 (SD 9.9) years in group 1 and 54.1 (SD 12.1) years in group 2. More female participants were present in group 1 (109/169, 64.5%) and group 2 (260/380, 68.4%) than male participants in the study. The average BMI of participants was 33.9 (SD 10.2) kg/m² for group 1 and 34.9 (SD 9.6) kg/m² for group 2. A mix of races and ethnicities were represented, with the majority of participants being White (128/169, 75.7% in group 1 and 283/380, 74.5% in group 2).

**Table 2:** Patient demographics.

Demographics			Cohort		
			Group 1 population (n=169)^a^		Group 2 population (n=380)^b^
**Age (years)**					
	Participants, n	169		379	
	Mean (SD)	58.1 (9.9)		54.1 (12.1)	
	Median (range)	58.0 (34.0-80.0)		55.0 (22.0-87.0)	
**Sex, n (%)**					
	Female	109 (64.5)		260 (68.4)	
	Male	60 (35.5)		118 (31.1)	
	Missing	0 (0)		2 (0.5)	
**Race and ethnicity, n (%)**					
	White	128 (75.7)		283 (74.5)	
	Black	27 (16)		56 (14.7)	
	Other	12 (7.1)		28 (7.4)	
	2 or more^c^	2 (1.2)		11 (2.9)	
	Missing	0 (0)		2 (0.5)	
**Height (cm)**					
	Participants, n	168		377	
	Mean (SD)	168.6 (10.3)		168.2 (10.4)	
	Median (range)	167.6 (149.9-198.1)		167.6 (144.8-198.1)	
**Weight (kg)**					
	Participants, n	169		377	
	Mean (SD)	96.4 (30.0)		98.7 (28.5)	
	Median (range)	90.9 (43.6-239.5)		94.5 (43.6-239.5)	
**BMI (kg/m²)**					
	Participants, n	168		376	
	Mean (SD)	33.9 (10.2)		34.9 (9.6)	
	Median (range)	31.8 (18.3-87.9)		33.1 (18.3-90.3)	

^a^Includes all participants who registered at least 11 of 14 routines in the app during the 1st and 12th study week, and in at least 8 of the 10 remaining study weeks.

^b^Includes all enrolled participants who completed the study and entered any routines (9 enrolled participants did not enter any routines).

**^c^**Participants who identified as 2 or more races and ethnicities.

### Adherence and Participant Satisfaction

Central to examining the core participant interactions with the app was the adherence of participants to record their daily routines (Table 3). Out of all 380 enrolled participants (group 2), a total of 248 (65.3%) participants recorded full BP routines 80% or more over the 12-week period and 322 (84.7%) participants recorded any data on 80% or more of the days over the 12-week period. Out of the 239 participants who remained in the study for the full 12-week observation period, 201 (84.1%) participants recorded full BP routines 80% or more over the 12-week period, and 227 (95%) participants of patients recorded any data on 80% or more of the days over the 12-week period. The number of study participants decreased from 380 at week 1 to 239 (62.9%) at week 12. Participants also had the option to continue for an additional 12 weeks resulting in an observational period of up to 23 weeks. The adherence to entry of routines for up to an additional 12 weeks was assessed (Multimedia Appendix 3), which looked at the total number of participants listed for each week, including only those participants who entered routines for the week. Of the 239 participants who completed the 12-week study period, 234 progressed to the optional 12-week extension period and started a 13th week. Furthermore, 155 participants progressed to week 14 and 99 participants continued to week 15. At this point, the number of participants dropped to 57 at week 16 and less than 10% of enrollees (30/380, 7.9% of participants) continued to enter data after week 17. However, it should be noted that the length of participation in the study was also dependent on the remaining time for recruitment and overall study completion; thus, some participants did not have the opportunity to participate in the extension period or could only participate in a time limited portion.

Participant feedback and satisfaction using the digital diary to record BP routines was assessed using the UEQ. The UEQ uses 26 questions, which are presented as a scale from 1 to 7 with a positive term and a negative term on each end of the scale (eg, annoying=1 to enjoyable=7). The 26 questions are grouped into 6 domains, which are attractiveness, perspicuity, efficiency, dependability, simulation, and novelty. A mean score for each domain is presented, whereby mean values for the domains can range from –3 to +3. Mean values between –0.8 and +0.8 are considered neutral, mean values above 0.8 are considered a positive evaluation, and mean values less than –0.8 are considered a negative evaluation. However, it should be noted that question number 26 from the UEQ that asks if users found the app to be “conservative or innovative” was missing from the dataset. Therefore, there are not any data present for this question, and it does not contribute toward the mean scores for the domain it belonged to (novelty).

**Table 3:** Participant compliance with remote data collection (number of routines^a^ recorded).

Analysis group		Full routine^a^	Full BP^b^ routine^c^	Any entry within a day
**Group 2** ^d^ **(n=380)**				
	Over 80% adherence, n (%)	241 (63.4)	248 (65.3)	322 (84.7)
	Median adherence (%), median (IQR)	87.1 (69.3-97)	88 (70.3-97)	100 (92.5-100)
**Participants who completed 12-week observation period (subset of group 2** ^d^ **, n=239)**				
	Over 80% adherence, n (%)	196 (82)	201 (84.1)	227 (95)
	Median adherence (%), median (IQR)	92.8 (83.9-97.6)	94 (84.4-98.2)	100 (98.8-100)

^a^A full routine consists of morning BP in triplicate along with medication summary, or evening BP in triplicate along with a symptom report.

^b^BP: blood pressure.

^c^A full BP routine consists of morning blood pressure in triplicate or evening blood pressure in triplicate.

^d^Includes all enrolled participants who completed the study and entered any routines (9 enrolled participants did not enter any routines).

**Figure 2:**
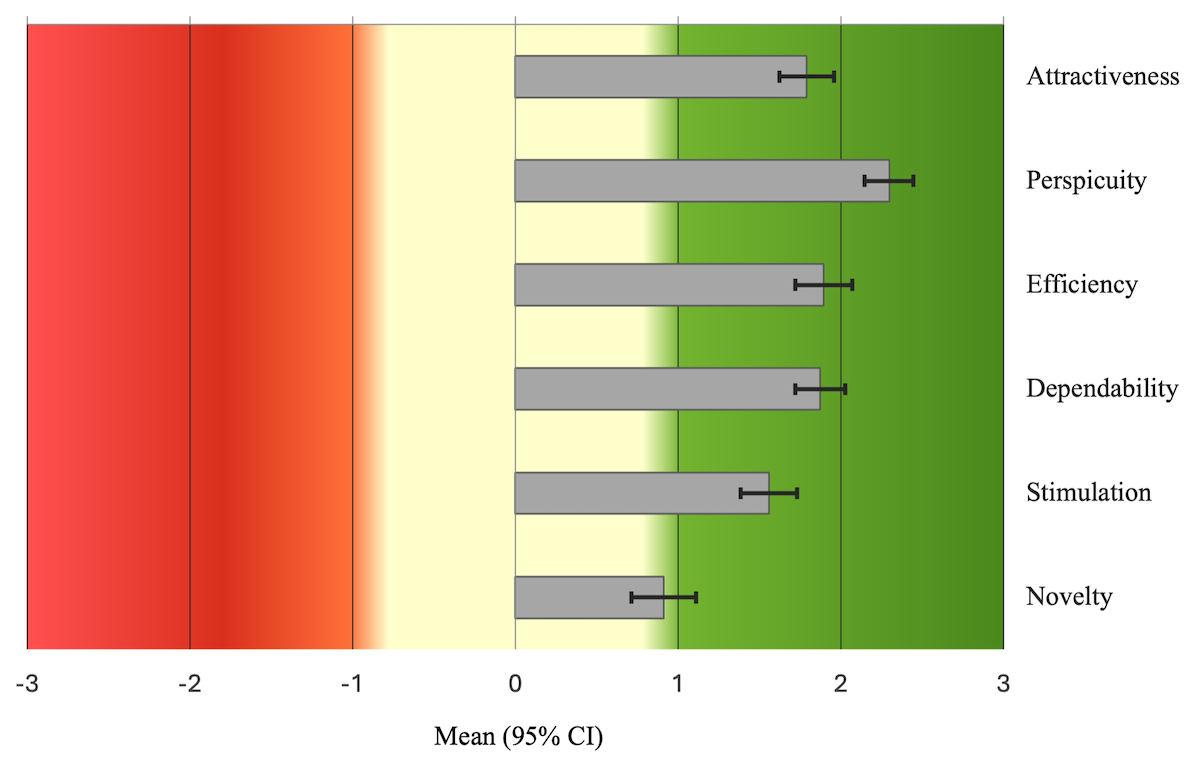
User Experience Questionnaire scores (all enrolled participants).

Almost all single-scored items of the UEQ averaged a positive evaluation (>0.8; only inventive to conventional scored lower [mean score 0.5]), with the majority scoring a very good evaluation (1.5 or above). Figure 2 shows the analysis of the 6 domains representing groups of single items within the UEQ. These results revealed very good to excellent evaluations for attractiveness (mean 1.79, SD 1.29), perspicuity (mean 2.30, SD 1.03), efficiency (mean 1.90, SD 1.43), dependability (mean 1.87, SD 1.09), and stimulation (mean 1.56, SD 1.41). The score for novelty was slightly lower (mean 0.91, SD 1.83) but was still a positive evaluation. The UEQ can be used to benchmark products against other apps, web pages, software, and social networks, with data coming from 21,175 persons involved in 468 studies regarding different products [7]. The digital app in this study was benchmarked as “excellent” (top 10% best results) for perspicuity, efficiency, and dependability and as “good” (10% of results are better and 75% of results are worse) for attractiveness and stimulation. Novelty scored an “above average” UEQ benchmark (25% of results are better and 50% of results are worse).

### BP Control

Changes in self-reported average systolic BP (SBP) and diastolic BP (DBP) during the observational period for group 1 and subgroups within this population are shown in Table 4. No significant changes in average SBP (mean –0.9, SD 10.7 mm Hg; *P*=.30) or DBP (mean –1.0, SD 7.9 mm Hg; *P*=.10) were detected between week 1 and week 12. Subgroup analysis by age (≥65 y and <65 y) and sex also showed no significant changes in BP during the 12-week period (all *P*>.05). Average levels for SBP (week 1: mean 129.5, SD 13.4 mm Hg; week 12: 128.6, SD 13.6 mm Hg) and DBP (week 1: 79.2, SD 9.9 mm Hg; week 12: 78.2, SD 10.8 mm Hg) were below UK and US hypertensive diagnostic thresholds.

Comparison of BP levels measured at week 1 and week 12 by race for group 1 showed no significant changes in SBP or DBP for Black participants or in SBP for White participants (all *P*>.05). A statistically significant change in average DBP between baseline and 12 weeks was observed for White participants (mean –1.6, SD 7.6 mm Hg; *P*=.02). No significant changes in SBP or DBP for participant groupings based on current antihypertensive medication (angiotensin-converting enzyme inhibitor [ACEi] or angiotensin receptor blocker [ARB], β-blockers, or calcium channel blockers [CCB]) between week 1 and week 12 were observed (all *P*>.05), except for a minor decrease in DBP (mean –2.1, SD 6.6 mm Hg; *P*=.01) observed in participants currently taking β-blockers.

Subgroupings of group 1 derived from hypertensive diagnostic thresholds (135/85 mm Hg and 140/90 mm Hg) revealed significant reductions at week 12 in comparison with week 1 in SBP (BP≥135/85 mm Hg group: mean –6.8, SD 10.3 mm Hg, *P*<.001; BP≥140/90 mm Hg group: –9.0, SD 10.8 mm Hg, *P*<.001) and DBP (BP≥135/85 mm Hg group: mean –3.8, SD 10.0 mm Hg, *P*=.02; BP≥140/90 mm Hg group: mean –5.4, SD 9.2 mm Hg, *P*=.008) for participants entering the study with BP equal to or higher that these thresholds. No changes in SBP or DBP over the 12-week period were observed in participants with average BP levels lower than these thresholds, except for a minor increase (mean 2, SD 9.8 mm Hg; *P*=.03) in SBP in participants joining the study with BP<135/85 mm Hg.

**Table 4:** Change in blood pressure by subgroup (group 1^a^).

Subgroup		Systolic BP^b^						Diastolic BP				
		Participants, n	Week 1, mean (SD)	Week 12, mean (SD)	Week 1-12 change, mean (SD)	*P* value	Participants, n		Week 1, mean (SD)	Week 12, mean (SD)	Week 1-12 change, mean (SD)	*P* value
**Overall population**		169	129.5 (13.4)	128.6 (13.6)	–0.9 (10.7)	.30	169		79.2 (9.9)	78.2 (10.8)	–1.0 (7.9)	.10
**Age group (years)**												
	≥65	51	126.5 (15.2)	126.4 (14.8)	–0.0 (9.7)	.99	51		74.6 (9.3)	73.4 (8.7)	–1.2 (5.8)	.16
	<65	118	130.8 (12.3)	129.5 (12.9)	–1.2 (11.2)	.24	118		81.2 (9.5)	80.2 (10.9)	–1.0 (8.7)	.23
**Sex**												
	Male	60	132.1 (13.9)	131.1 (15.0)	–1.0 (11.6)	.52	60		79.6 (9.7)	79.1 (11.2)	–0.5 (7.6)	.64
	Female	109	128.0 (12.9)	127.2 (12.6)	–0.8 (10.3)	.42	109		79.0 (10.0)	77.7 (10.5)	–1.3 (8.1)	.09
**Race**												
	White	128	129.7 (13.7)	128.5 (14.1)	–1.1 (11.3)	.27	128		78.7 (9.7)	77.1 (9.9)	–1.6 (7.6)	.02
	Black	27	128.0 (11.4)	128.1 (10.7)	0.1 (8.7)	.94	27		81.2 (9.8)	81.3 (10.4)	0.1 (7.8)	.96
**HBP** ^c^ **(United States)**												
	BP ≥140/90 mm Hg at entry	33	150.2 (7.1)	141.2 (12.7)	–9.0 (10.8)	<.001	24		95.3 (7.5)	89.9 (9.3)	–5.4 (9.2)	.008
	BP <140/90 mm Hg at entry	136	124.4 (9.0)	125.5 (11.9)	1.1 (9.8)	.19	145		76.5 (7.4)	76.3 (9.7)	–0.3 (7.5)	.65
**HBP (United Kingdom)**												
	BP ≥135/85 mm Hg at entry	55	145.0 (8.5)	138.3 (11.8)	–6.8 (10.3)	<.001	40		92.2 (7.0)	88.5 (10.0)	–3.8 (10.0)	.02
	BP <135/85 mm Hg at entry	114	121.9 (7.6)	123.9 (11.8)	2.0 (9.8)	.03	129		75.2 (6.6)	75.0 (8.8)	–0.2 (7.0)	.79
**Drug Class**												
	ACE^d^ inhibitors or AR^e^ blockers	110	128.9 (13.0)	128.9 (13.5)	0.0 (10.7)	.99	110		8.9 (10.0)	78.4 (11.8)	–0.5 (8.6)	.56
	β-blockers	70	131.5 (15.4)	128.9 (15.4)	–2.5 (11.1)	.06	70		80.0 (11.0)	77.9 (10.0)	–2.1 (6.6)	.01
	Calcium channel blockers	55	129.7 (12.0)	128.3 (11.1)	–1.4 (11.1)	.35	55		79.1 (9.0)	79.1 (10.8)	0.1 (7.1)	.93

^a^Includes all participants who registered at least 11 of 14 routines in the app during the 1st and 12th study week, and in at least 8 of the 10 remaining study weeks.

^b^BP: blood pressure.

^c^HBP: home blood pressure.

^d^ACE: angiotensin-converting enzyme.

^e^AR: angiotensin receptor.

### Antihypertensive Medication Use

**Figure 3:**
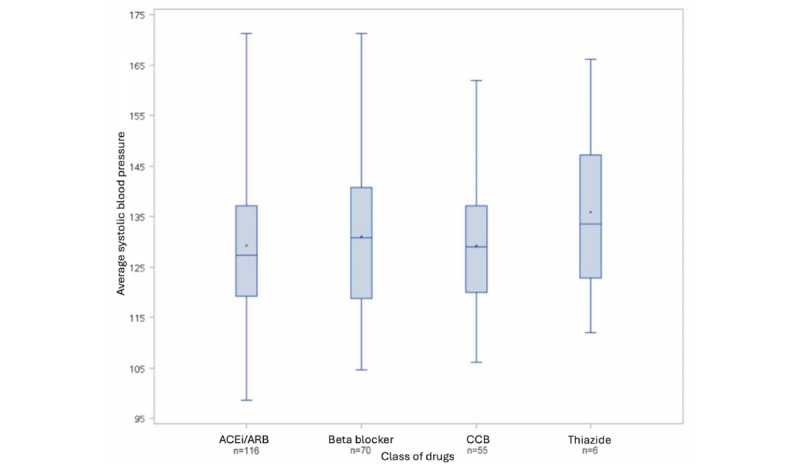
Average systolic blood pressure for each drug combination group (group 1 population). ACEi: angiotensin-converting enzyme inhibitor; ARB: angiotensin receptor blocker; CCB: calcium channel blocker.

**Figure 4:**
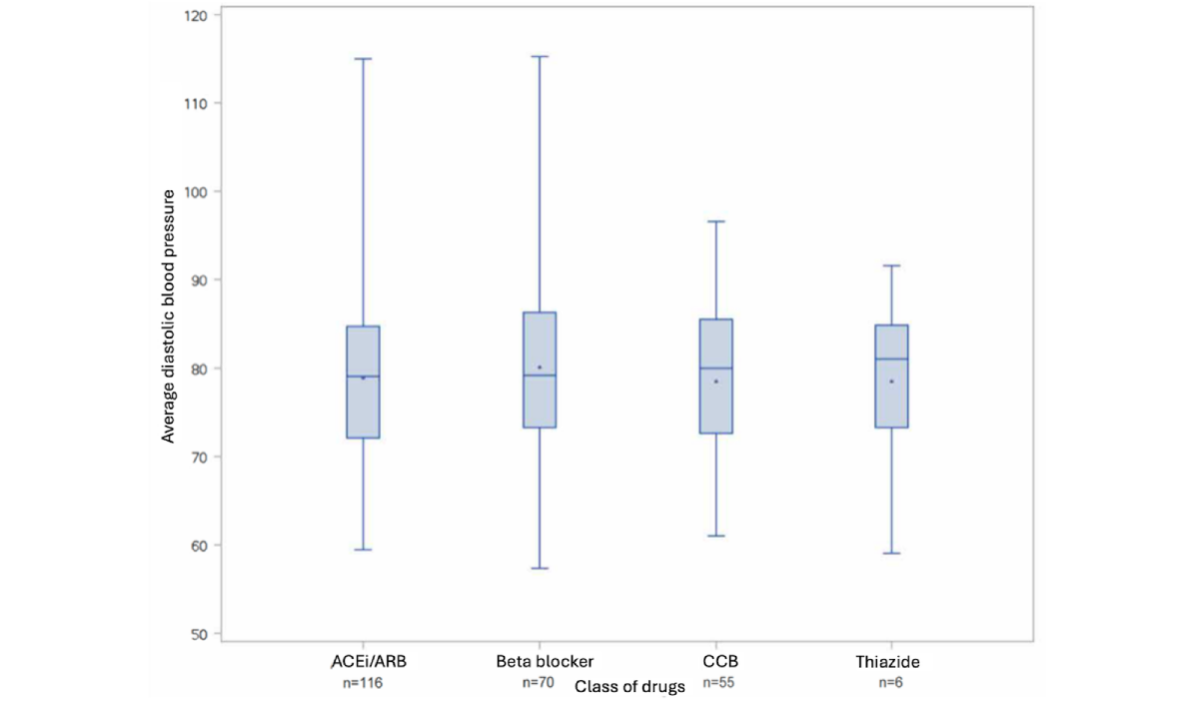
Average diastolic blood pressure for each drug combination group (group 1 population). ACEi: angiotensin-converting enzyme inhibitor; ARB: angiotensin receptor blocker; CCB: calcium channel blocker.

Antihypertensive medication use was examined for participants analyzed in group 1 (which includes all participants who registered at least 11 of 14 routines in the app during the 1st and 12th study week, and in at least 8 of the 10 remaining study weeks). No statistically significant differences in average SBP and DBP were seen for participants grouped by their antihypertensive medications (ACEi or ARB, β-blockers, and CCBs; all *P*>.05; Figures 3 and 4). Over the 12-week observational period, a significant but minor reduction in DBP (mean –2.1, SD 6.6 mm Hg; *P*=.01) in those participants currently taking β-blockers was observed (Table 3). ACEi or ARB were the most common antihypertensive medication taken (n=116), with smaller numbers of participants taking β-blockers (n=70), CCBs (n=55), or thiazide-type diuretics (n=6; Figure 3).

**Figure 5:**
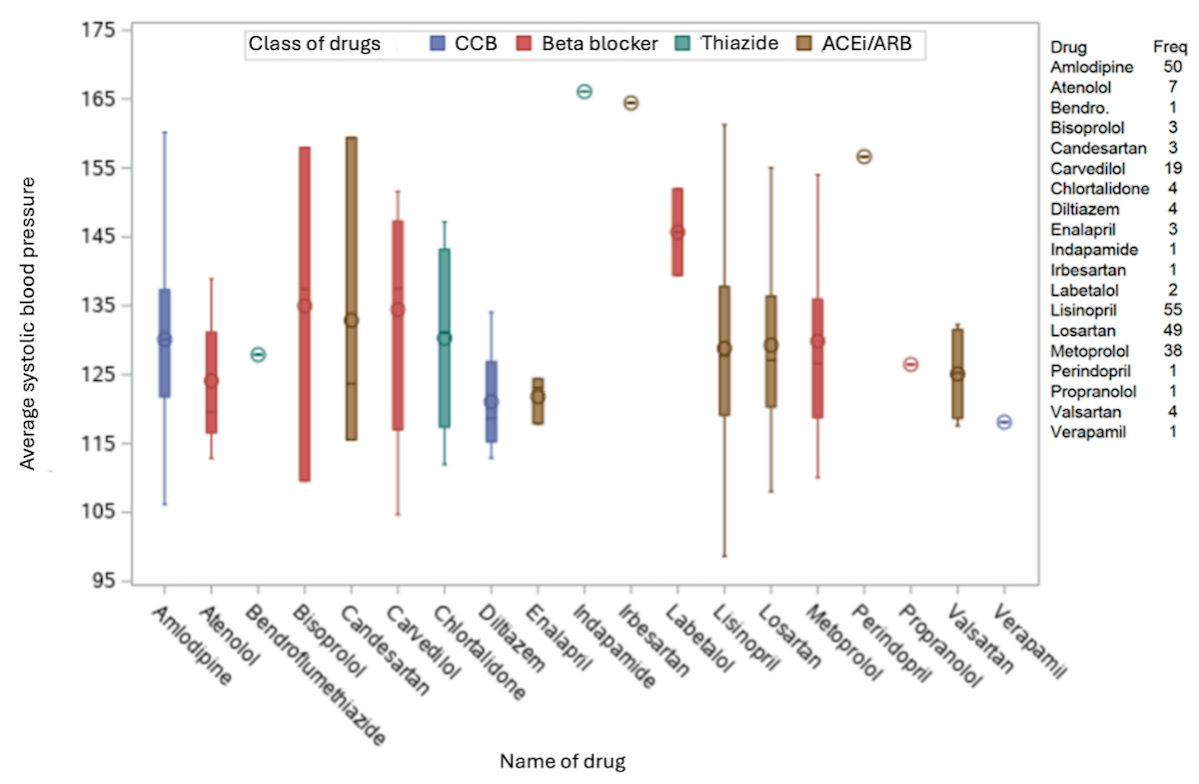
Average systolic blood pressure for each drug (group 1 population). ACEi: angiotensin-converting enzyme inhibitor; ARB: angiotensin receptor blocker; CCB: calcium channel blocker; freq: frequency.

**Figure 6:**
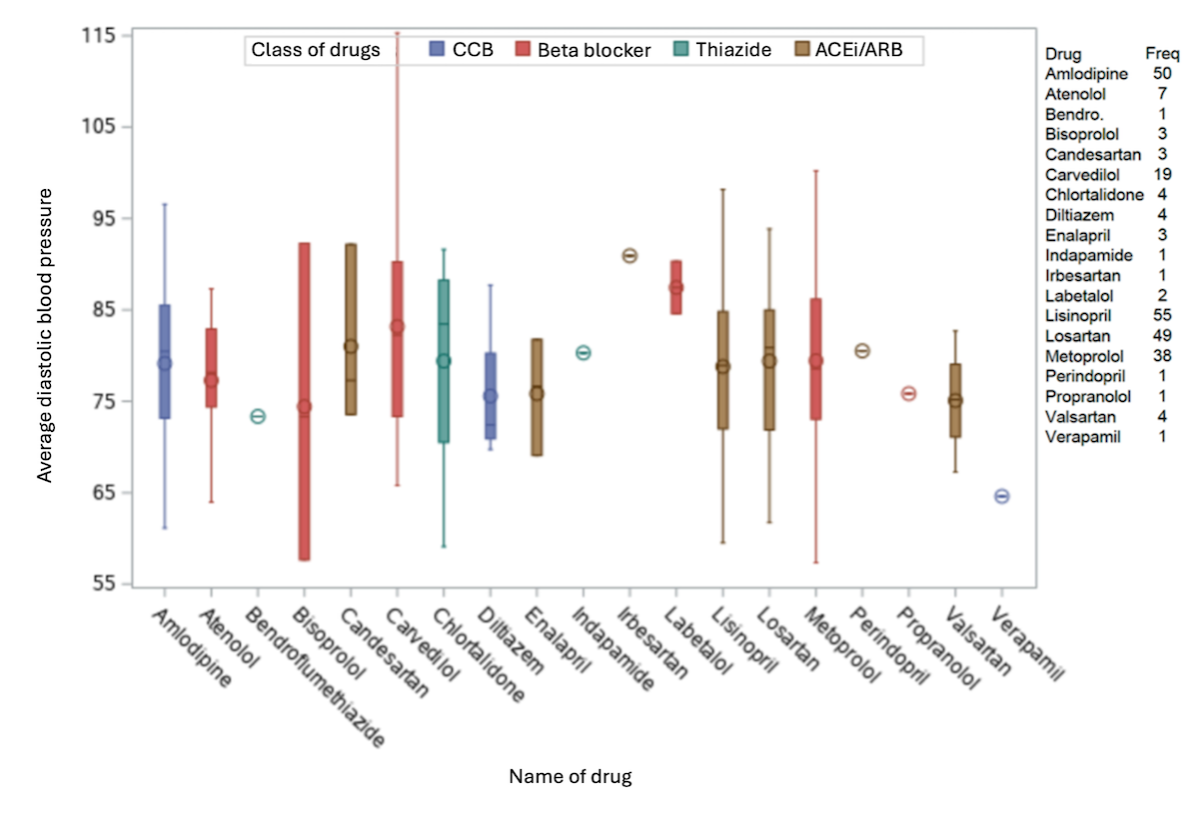
Average diastolic blood pressure for each drug (group 1 population). ACEi: angiotensin-converting enzyme inhibitor; ARB: angiotensin receptor blocker; CCB: calcium channel blocker; freq: frequency.

Further categorization of these participants by current antihypertensive medication revealed no statistically significant differences in average SBP and DBP (Figures 5 and 6). The most commonly taken antihypertensive medications in this population included lisinopril (ACEi; n=55), amlodipine (CCB; n=55), losartan (ARB; n=49), and metoprolol (β-blocker; n=38).

### COVID-19 Symptoms

An analysis of emergent symptoms of SARS-CoV-2 infection, including but not limited to fatigue, high temperature, persistent dry cough, chest pain, and shortness of breath, in all enrolled participants was performed. This revealed very low levels of symptoms related to infection with COVID-19, with only 3 participants experiencing these symptoms. Due to insufficient numbers of participants experiencing COVID-19 symptoms, associations between BP medication and BP level with SARS-CoV-2 infection and COVID-19 outcomes could not be assessed.

### Adverse Events

All enrolled participants (n=398) were included in the safety population. There were no AEs attributable to the smartphone app, SAEs, or deaths recorded in this study.

## Discussion

### Principal Findings

Our study successfully deployed a smartphone app used for monitoring daily BP, medication, and side effects in a community setting within the United States, and almost all participants (380/389, 98%) downloaded and entered BP routines in week 1. A high proportion of participants remained in the study for the full 12-week observation period and entered full BP routines into the digital app 80% or more of the time. Participants reported their experience of using the smartphone app using the UEQ, giving an overall positive evaluation, whereby the app was benchmarked “excellent” and “above average” for almost all domains. Highly adherent participants with hypertension demonstrated well-controlled BP, with no significant changes in average SBP or DBP between week 1 and week 12. Participants were able to record BP medications and symptoms of SARS-CoV-2 infection. No AEs attributable to use of the smartphone app were reported during the observational period.

The study population had a broad age range, between 24 and 87 (mean 54.1) years. There were more female than male participants enrolled (260/380, 68.4%), and analysis of race and ethnicity showed the population consisted of 74.5% (283/380) White participants, 14.7% (56/380) Black participants, 7.4% (28/380) participants who reported “other” for their race and ethnicity, and 2.9% (11/380) participants who reported their race and ethnicity as “2 or more.” Any participant who did not report as being White, Black, or having 2 or more races or ethnicities were required to enter their ethnicity as “other.”

According to the 2020 US decennial census, out of 331,449,281 people, there were 204,277,273 (61.6%) White (alone) individuals; 41,104,200 (12.4%) Black individuals; and 33,848,942 (10.2%) individuals who reported “2 or more” for their race and ethnicity [10]. In comparison with the reported demographics of the Unites States, we observed a higher percentage of White and Black participants enrolled into the study. However, a recent report describing racial and ethnic demographics of adults with hypertension in the United States reported 79,910,050 individuals with hypertension, of which 66.4% (51,03,185) were White and 14.4% (11,061,686) were Black [11]. The percentage of Black participants in our study is representative of the proportion of Black patients with hypertension in the United States; however, we observed a higher proportion of White participants than the proportion of White patients with hypertension in the United States. Our study observed 28 (7.4%) out of 380 participants who reported their race and ethnicity as “other,” which included any individuals did not identify as Black, White, or having “2 or more” races and ethnicities; so, we did not focus on other racial and ethnic groups specifically. The average BMI of the study population was 34.9 kg/m². These results indicate that, on average, the population was clinically obese, which are consistent with levels of obesity reported for US-based adults with hypertension [11].

CLM successfully modified an existing app and deployed it for use in the study. The patient experience and data collection components that had been developed as part of an early platform concept proved straightforward to adapt and extend for this study, building confidence that the goal of rapidly developing an adaptable multitherapeutic technology for patient data collection and treatment is viable. During pandemic conditions, the ability to quickly respond with digital patient support and assessment tools is critical [12]. Symptom collection proved straightforward by reusing user interface components from the early platform that allowed patients to rate their symptoms using a visual analog scale (VAS). The symptom component was designed to present a configurable and customizable list of symptoms to the patient, which allows the data to be collected, stored, and presented. A similar approach was taken with the hypertension drug list. The patient experience component required only simple adaptation as it was directly suitable for adoption in this study. The changes were simple, such as updating the app name or contact information presented to patients. This provided reassuring evidence that a common set of user interaction components, supported by an underlying platform, can be viable in a digital health care setting involving mobile devices.

In this study, we used the smartphone app to record daily BP measurements in patients with hypertension. Telemonitoring is another method that is used for routine BP monitoring in hypertension [13]. Industry standards and other clinical studies have shown that 39% to 72% of patients report their BP 80% or more of the time using this method [14-17]. In our study, 248 (65.3%) out of 380 participants (group 2) showed high compliance for entering daily BP routines into the CLM digital diary (entering full BP routines on 80% or more of days). Compliance to entering daily BP routines was even higher in the 239 who completed the full 12 weeks, with 201 (84.1%) entering full BP routines over 80% of the time. While overall adherence levels may have been affected by the daily requirement to enter 2 routines (morning and evening) within a limited time window, recording morning and evening routines each day reflects best practice. Nonetheless, these results demonstrate that our test smartphone app meets industry norms for adherence to routine BP self-monitoring. It should be noted that the digital app development deployed in this study was not designed for high engagement and still achieved these industry standards. Future work will focus on refinement and incorporation of engagement enhancing features as part of product development.

Participant satisfaction of the CLM smartphone app for remote BP monitoring was assessed using the validated UEQ. The app scored an overall positive evaluation. Analysis of UEQ domains consisting of several questionnaire items, revealed that the app scored an excellent evaluation for perspicuity and very good evaluations for attractiveness, dependability, efficiency, and stimulation. A lower but still positive evaluation was given for novelty. Data from another hypertension study, PERSONAL-COVIDBP, in the United Kingdom has shown similar outcomes for user experience [18,19]. Overall, these results suggest very high levels of participant satisfaction of this smartphone app for daily monitoring of BP.

The UEQ can be used to benchmark products against other apps, web pages, software, and social networks, with data coming from 21,175 persons involved in 468 studies regarding different products [7]. Here, we show that our smartphone app for monitoring BP benchmarked “excellent” for domains of perspicuity, efficiency, and dependability and “good” for domains of attractiveness and stimulation. Novelty was benchmarked at “above average.” Again, these results indicate that despite the app being a prototype, a very high level of participant satisfaction was observed. Further app development work at CLM will include a particular focus on novelty, attractiveness, and stimulation.

### Clinical Outcomes

Through remote BP monitoring, we demonstrated that in a population of people with hypertension, self-reported BP in study completers was stable over a 3-month period with no significant changes in average BP (129.5/79.2 mm Hg at baseline, 128.6/78.2 mm Hg at week 12). These levels are below the established hypertensive diagnostic thresholds (United Kingdom: 135/85 mm Hg [20] and United States: 130/80 mm Hg [21]). These thresholds represent treatment targets for BP and as such, demonstrate that BP levels in this study population on average are being effectively managed. Although we do acknowledge that these results are based on self-reported data and that direct measurement might have resulted in higher BP data.

Although subgroup analysis showed that there was no difference in our BP outcomes across age and sex, significant reductions in SBP and DBP were observed for those participants who entered this study with baseline BP levels higher than the established hypertensive diagnostic thresholds for the United States and United Kingdom (Table 4). As this was an observational study with no drug intervention, these findings may have in part been a result of remote BP monitoring having a positive impact on BP control, when used in conjunction with standard of care antihypertensive medications. However, it is possible that a lowering of BP during the observational period could be due to other factors such as the Hawthorne Effect or regression to the mean, and in some cases, perhaps patients were at the early stages of their antihypertensive treatment. Nonetheless, our results are in line with the demonstrated effectiveness of BP self-monitoring, combined with other interventions, to lower BP in patients with high BP levels [22-24]. Self-monitoring of BP is thought to be advantageous as it can provide better estimation of underlying BP, increase adherence to medications, and reduce the need for clinic monitoring. In the future, remote BP monitoring would facilitate drug optimization through the development of more sophisticated electronic mobile apps.

According to American College of Cardiology and American Heart Association treatment guidelines for high BP, the first-line antihypertensives used to treat hypertension include ACEi, ARBs, CCBs, and thiazide-type diuretics [21]. After the Joint National Committee guidelines 8 in 2014, β-blockers have been considered secondary agents. Accordingly, this population of hypertensive patients based in the United States were seen taking 1 or more of these antihypertensive medications. Analysis of BP levels with respect to participant-reported antihypertensive drugs revealed no difference in levels of SBP and DBP reported for the participants taking thiazide-type diuretics, CCBs, ACEi, or ARBs. Similar results were seen for analyses of single antihypertensive drugs. We did however observe a minor decrease in DBP (mean 2.1, SD 6.6 mm Hg; *P*=.01) in those participants currently taking β-blockers at week 12.

### Strengths and Limitations

The strengths of this study were the rapid set up of a decentralized study during the COVID-19 pandemic, which involved the rapid modification and deployment of a smartphone app for monitoring BP, BP medication use, and symptoms of SARS-CoV-2 infection. Another strength of this research includes the large sample size and the accurate representation of Black participants in our study against the proportion of Black patients with hypertension in the United States. The low dropout rates also eliminated the need to correct for missing data. However, a limitation is that the study did not specifically look at Hispanic or Latino as well as Asian patients who have been reported under the “other” category of race and ethnicity; therefore, analysis specifically on these groups could not be performed.

Another limitation of this study was that data entry, adherence, and participant engagement rates may have been influenced by (1) participants being incentivized for entry of data into the smartphone app and (2) lockdown social distancing measures, which meant that patients with hypertension were at home without other activities occupying their time. Thus, study routines would have been less interrupted, possibly resulting in enhanced habit formation. Incentives were kept to a minimum, with the monetary value low to temper this limitation. As part of the UEQ, question-26 data were missing, which asked whether users considered the app to be “conservative or innovative.” As these data are missing and could have affected the mean score for the novelty domain in a positive or negative manner, it is a further limitation of the study.

Low incidence of symptoms of COVID-19 in the hypertensive study population was another limitation to this study. This meant that we were unable to examine associations between BP levels or BP antihypertensive drug use and the incidence of SARS-CoV-2 infection and severity of COVID-19 outcomes.

In our study, we evaluated changes in BP in a cohort compliant with BP data capture so we could accurately assess their BP levels. It is possible that those who were not as compliant with BP data capture may be different in some ways to this adherent population; however, as it would be less reliable to analyze BP in those with low BP data capture, it would be hard to make that assessment.

### Conclusions

We successfully deployed and tested a personalized electronic record in the form of a smartphone app in participants with primary hypertension for routine monitoring of BP in a community setting within the United States. The patient population engaged with the app and provided highly positive feedback. However, novelty features and additional engagement features beyond data monitoring should be considered to bolster patient adherence. This study also demonstrated no safety implications regarding the use of the smartphone app. These are important components for the provision of individualized and targeted treatment for hypertension.

## Appendix

Multimedia Appendix 1 Interface of the digital smartphone app.

Multimedia Appendix 2 System diagram.

Multimedia Appendix 3 Length of study participation during weeks 1 to 23 (all enrolled participants).

## Abbreviations

ACEi: angiotensin-converting enzyme inhibitor
AE: adverse event
ARB: angiotensin receptor blocker
BP: blood pressure
CCB: calcium channel blocker
CLM: Closed Loop Medicine
CRO: contract research organization
DBP: diastolic blood pressure
SAE: Serious Adverse Event
SBP: systolic blood pressure
UEQ: User Experience Questionnaire
VAS: visual analog scale
