# Supplementary material for: Personalized Smartphone-Enabled Assessment of Blood Pressure and Its Treatment During the SARS-CoV-2 COVID-19 Pandemic in Patients From the CURE-19 Study: Longitudinal Observational Study
Source: JMIR Mhealth Uhealth. 2024 Dec 3;12:e53430. doi: 10.2196/53430 (PMC11653031; doi:10.2196/53430)
Supplement: Multimedia Appendix 1 [file mhealth_v12i1e53430_app1.docx]

| Blood pressure monitoring | |
| --- | --- |
| 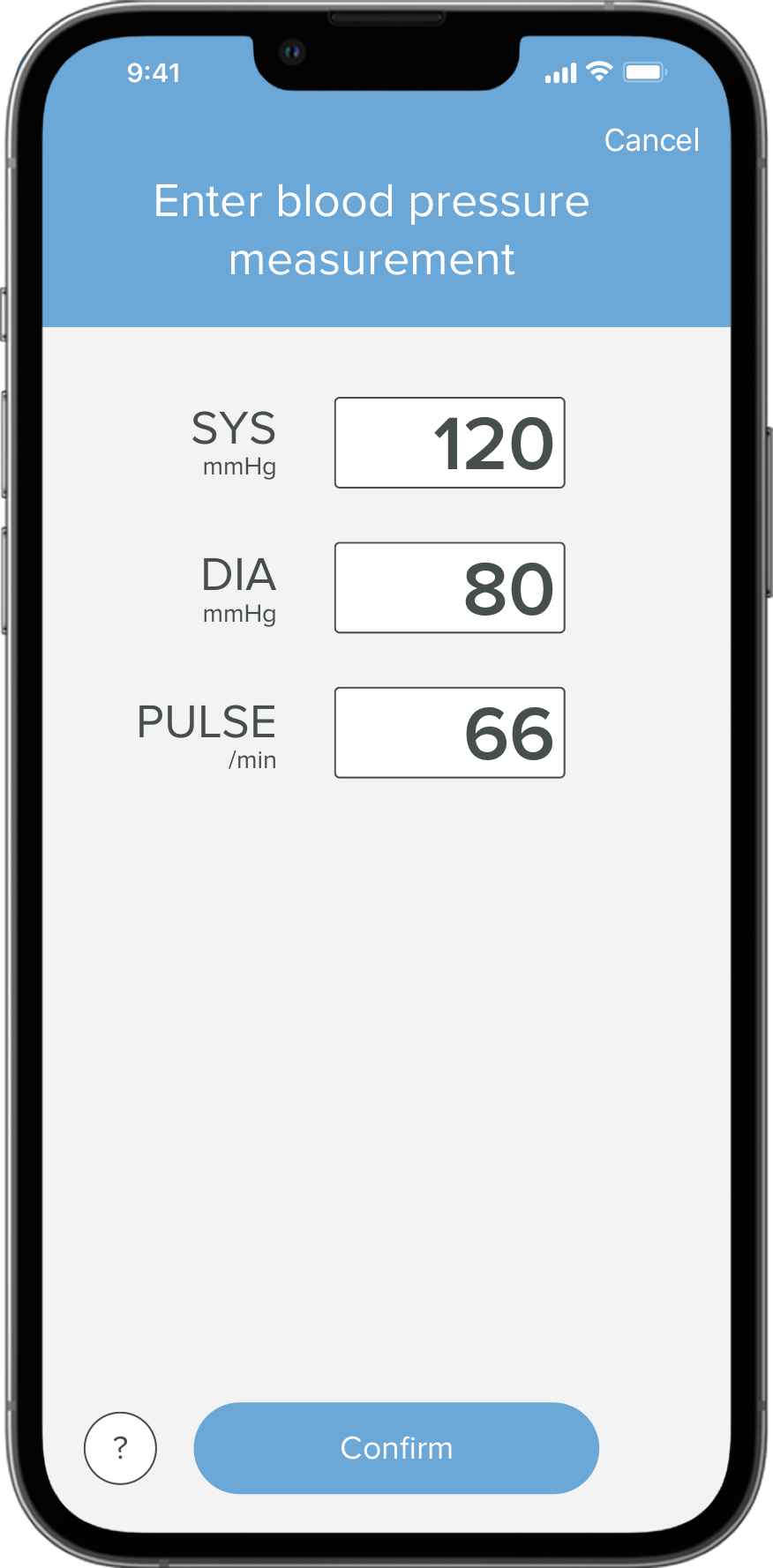 | 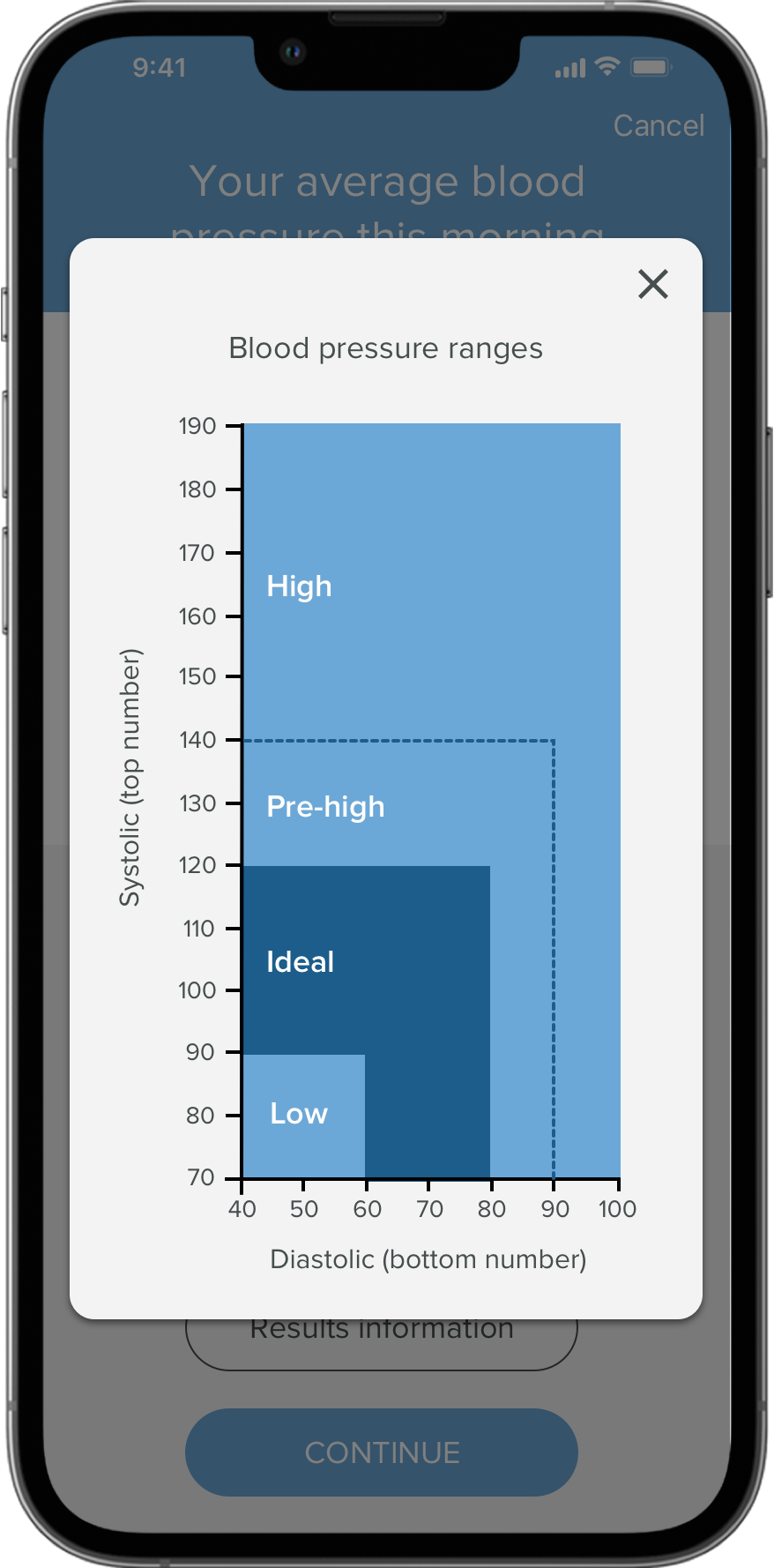 |
| COVID-19 symptom monitoring | |
| 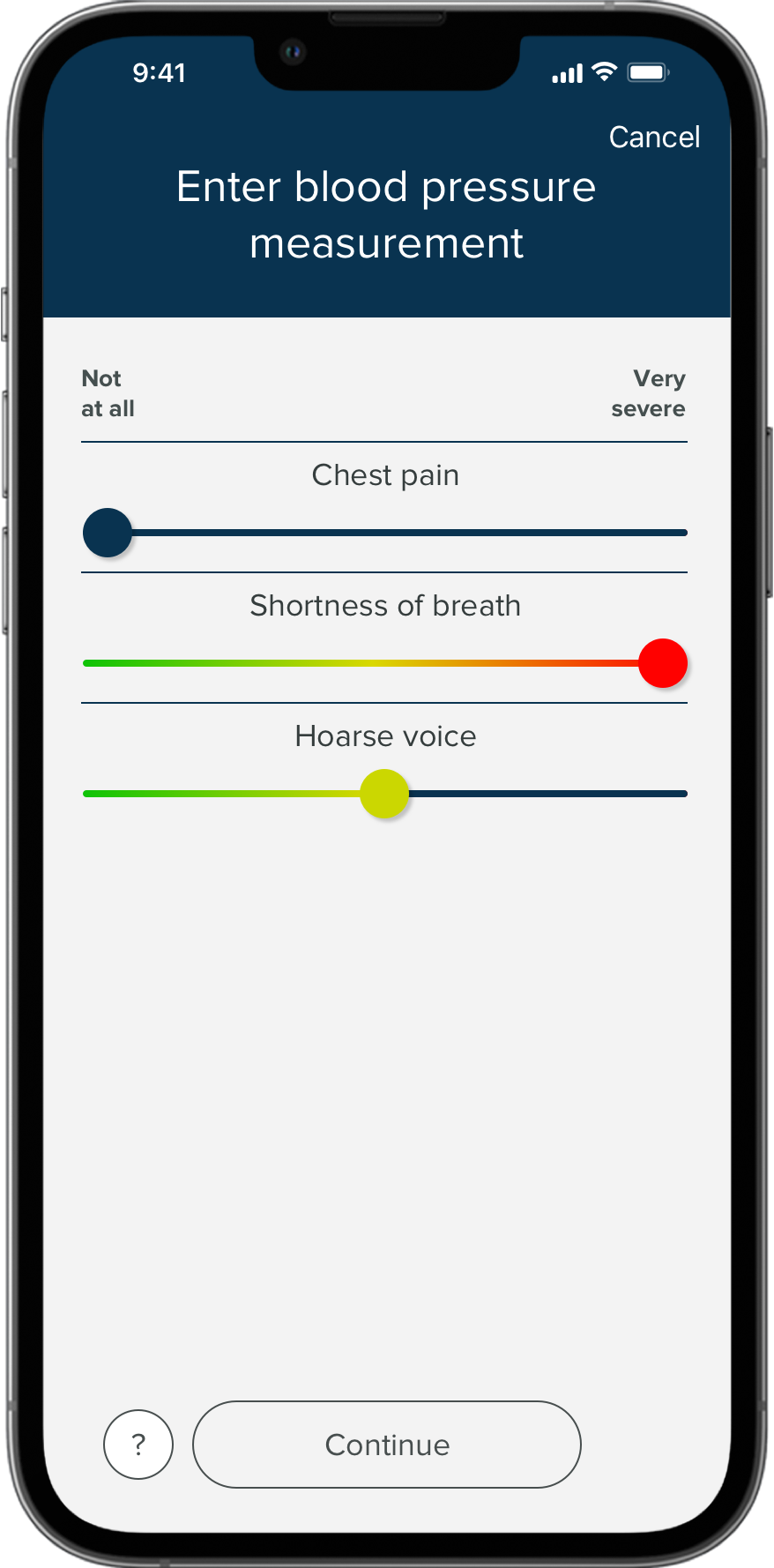 | 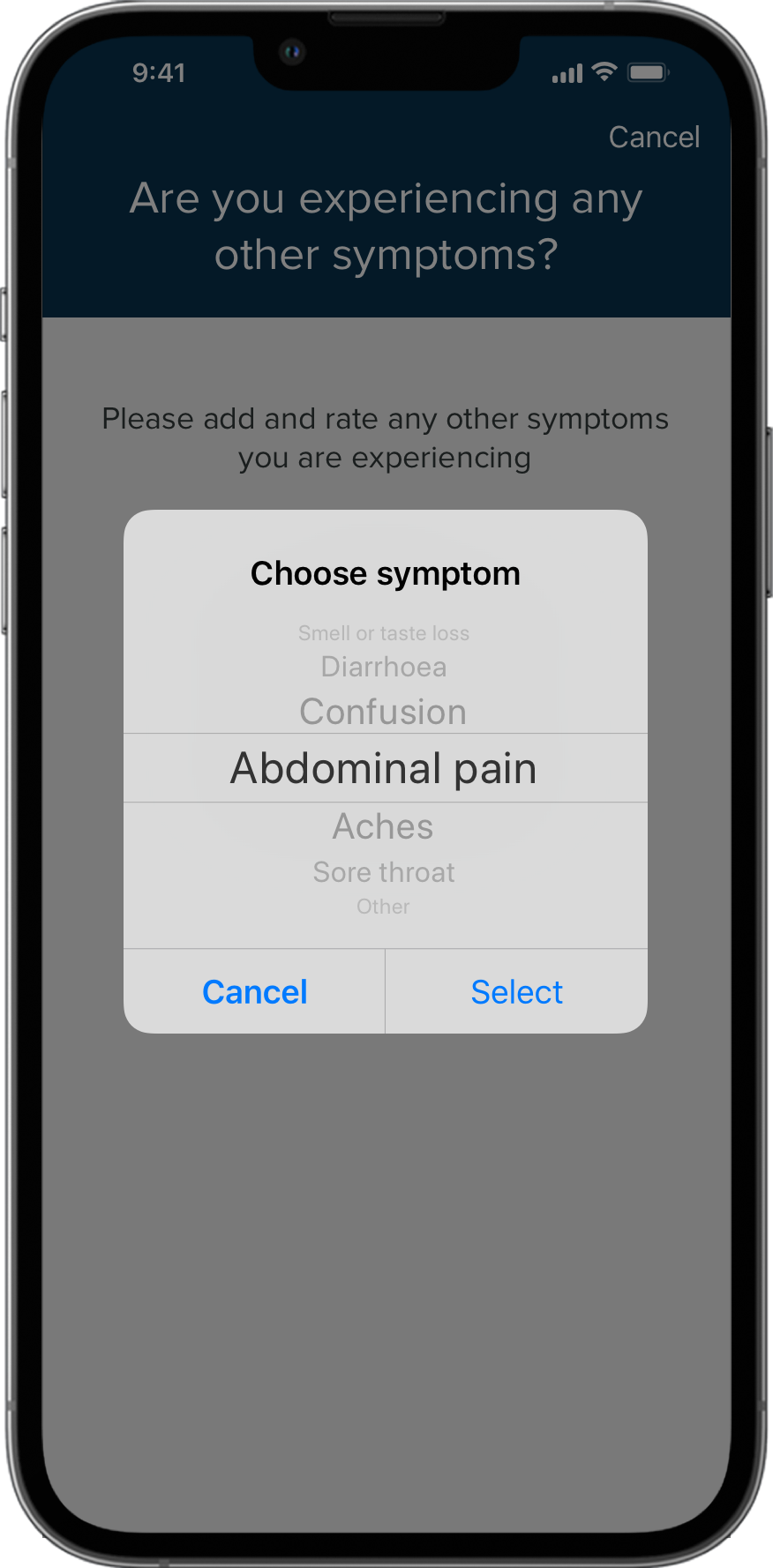 |

###

| Hypertension medication monitoring | |
| --- | --- |
| 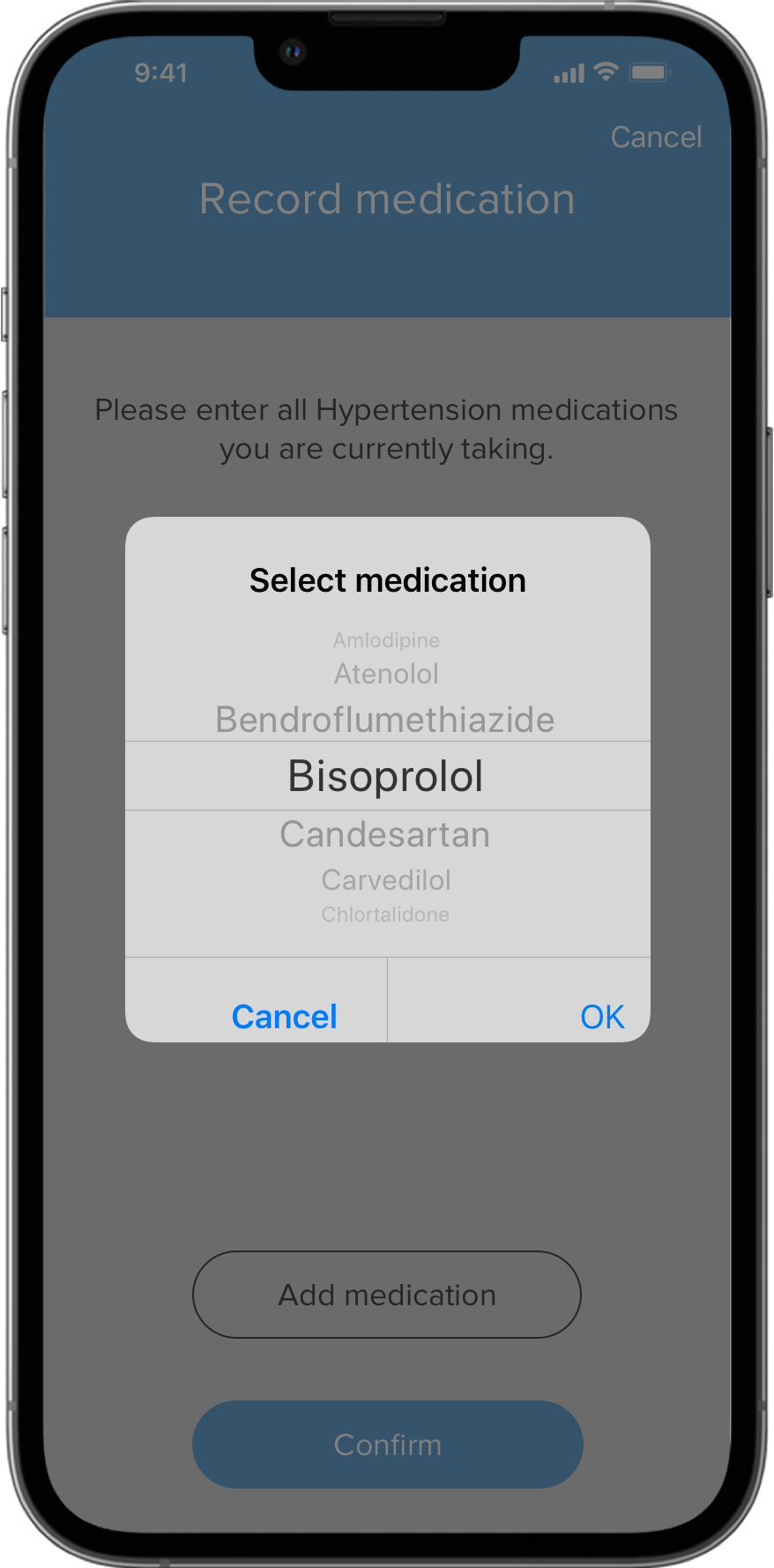 | **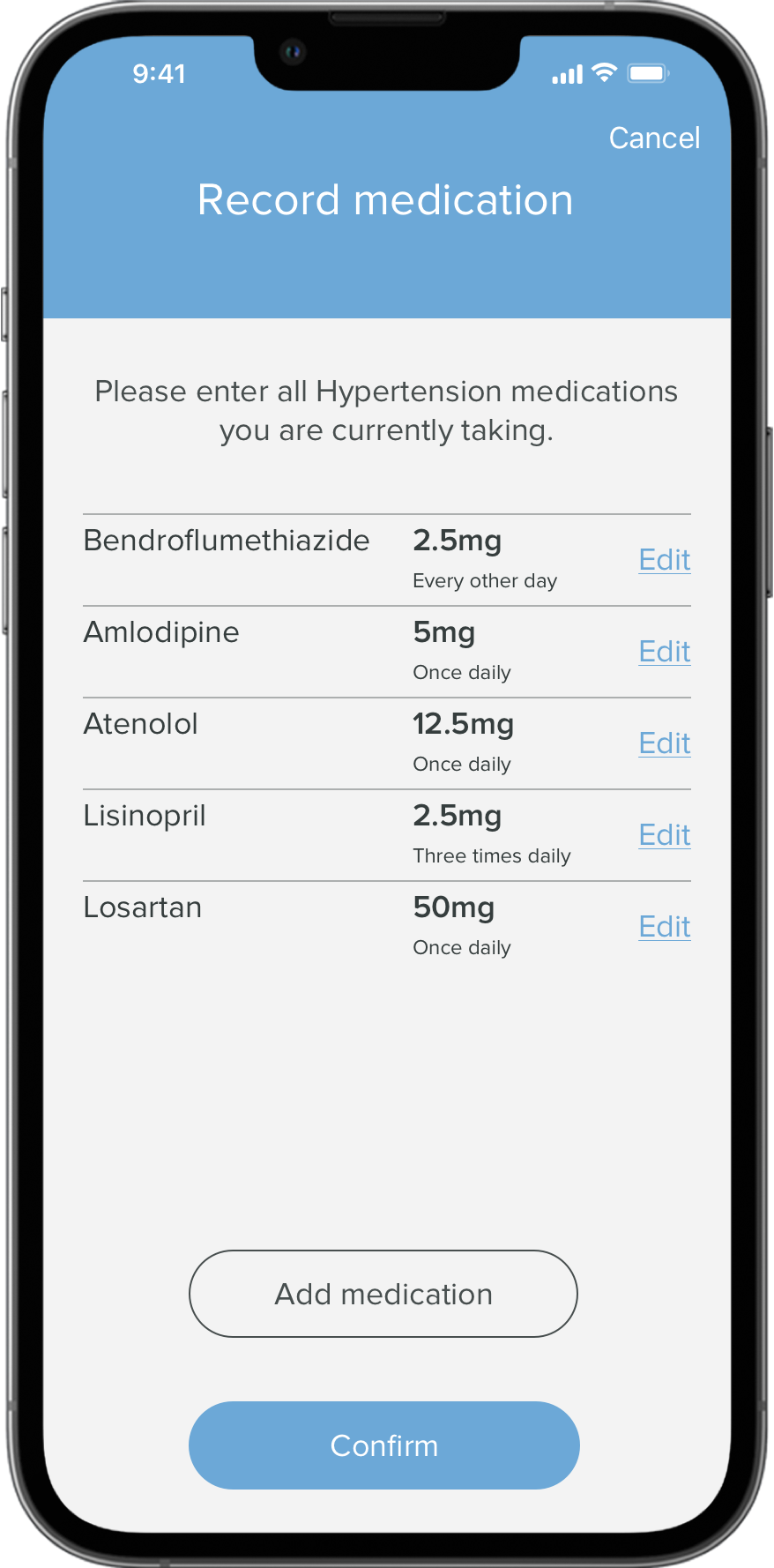** |
| Blood pressure measurement demonstration | |
| 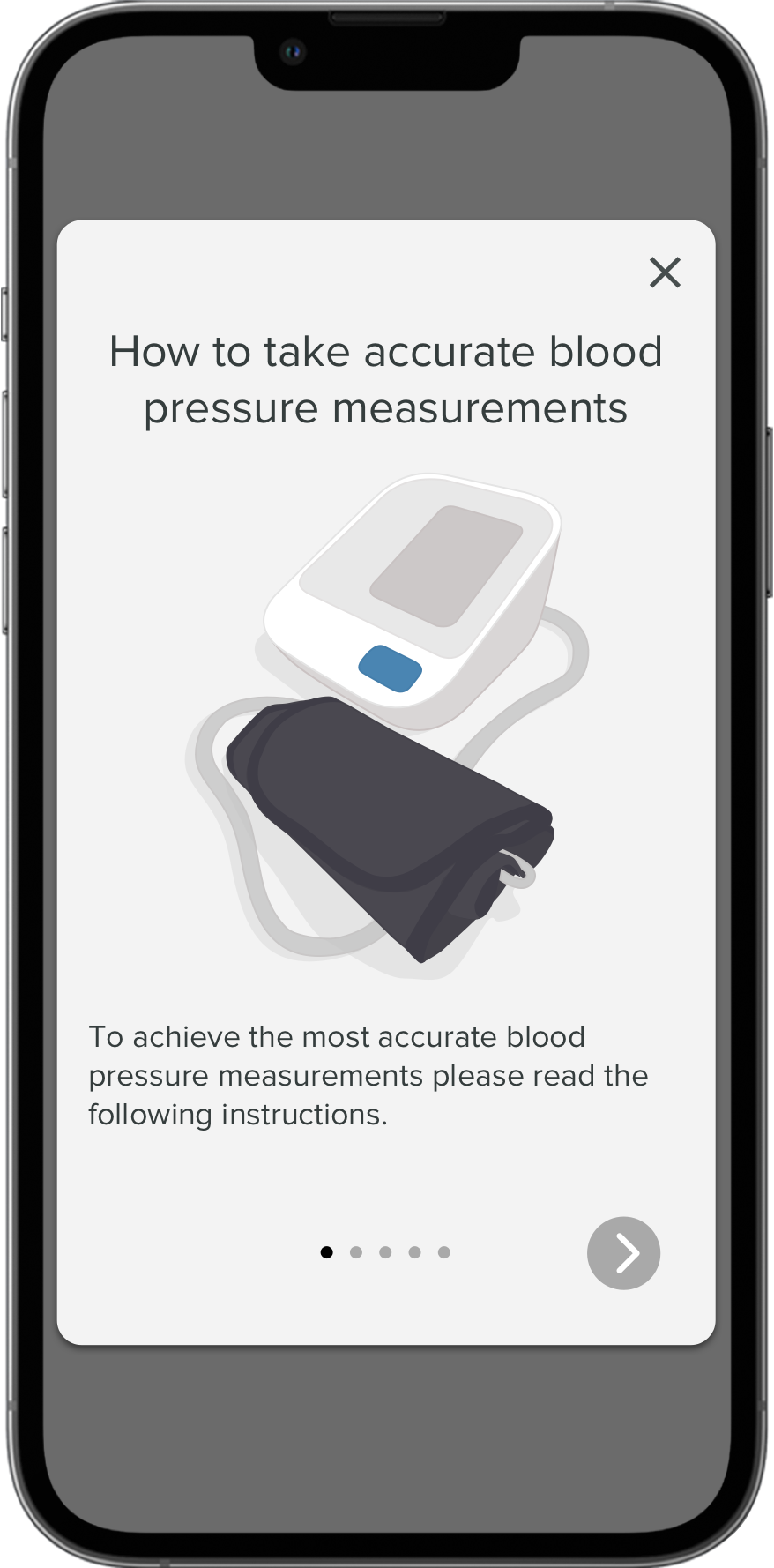 | **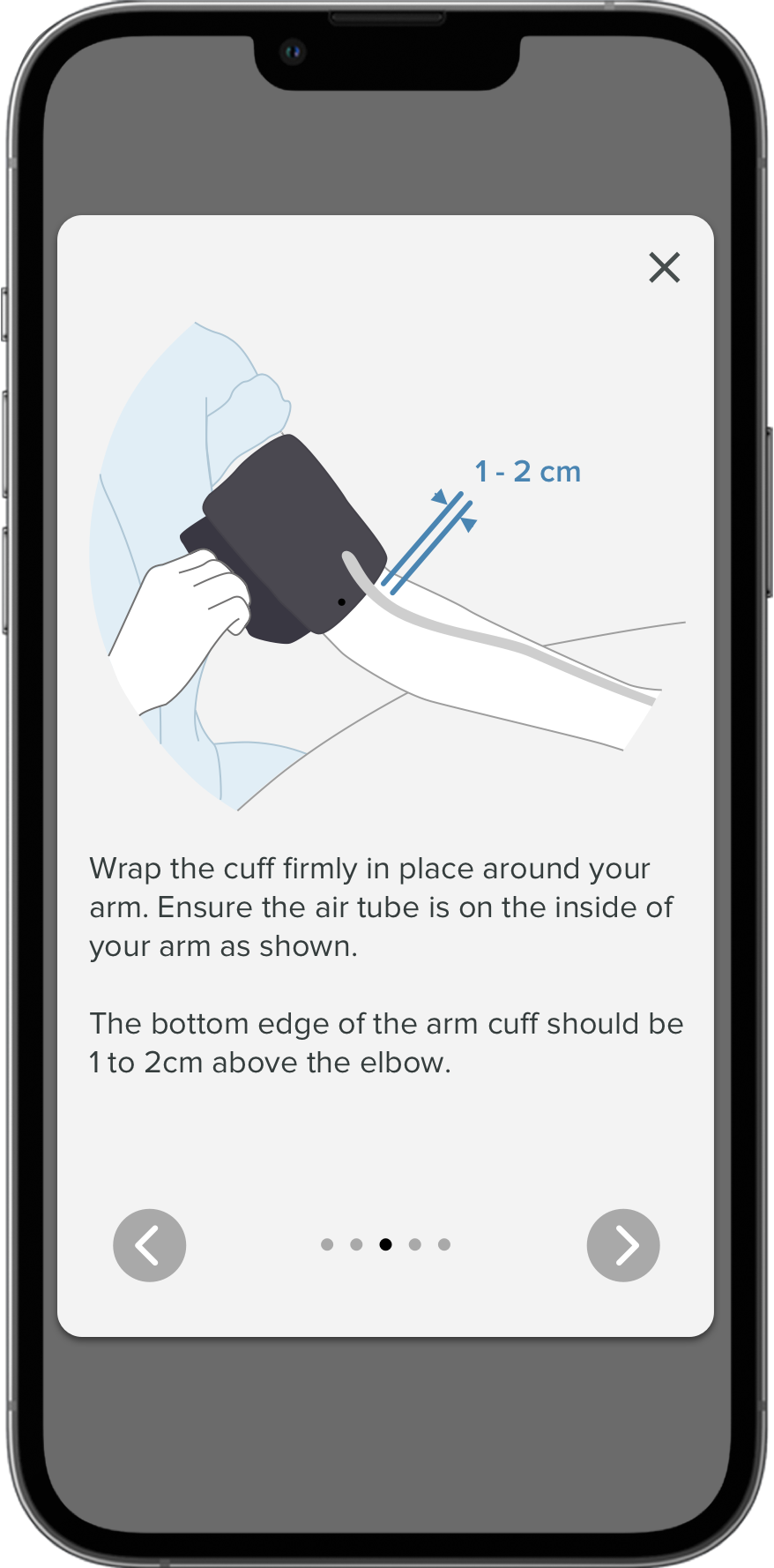** |

| COVID - 19 symptom Information | |
| --- | --- |
| 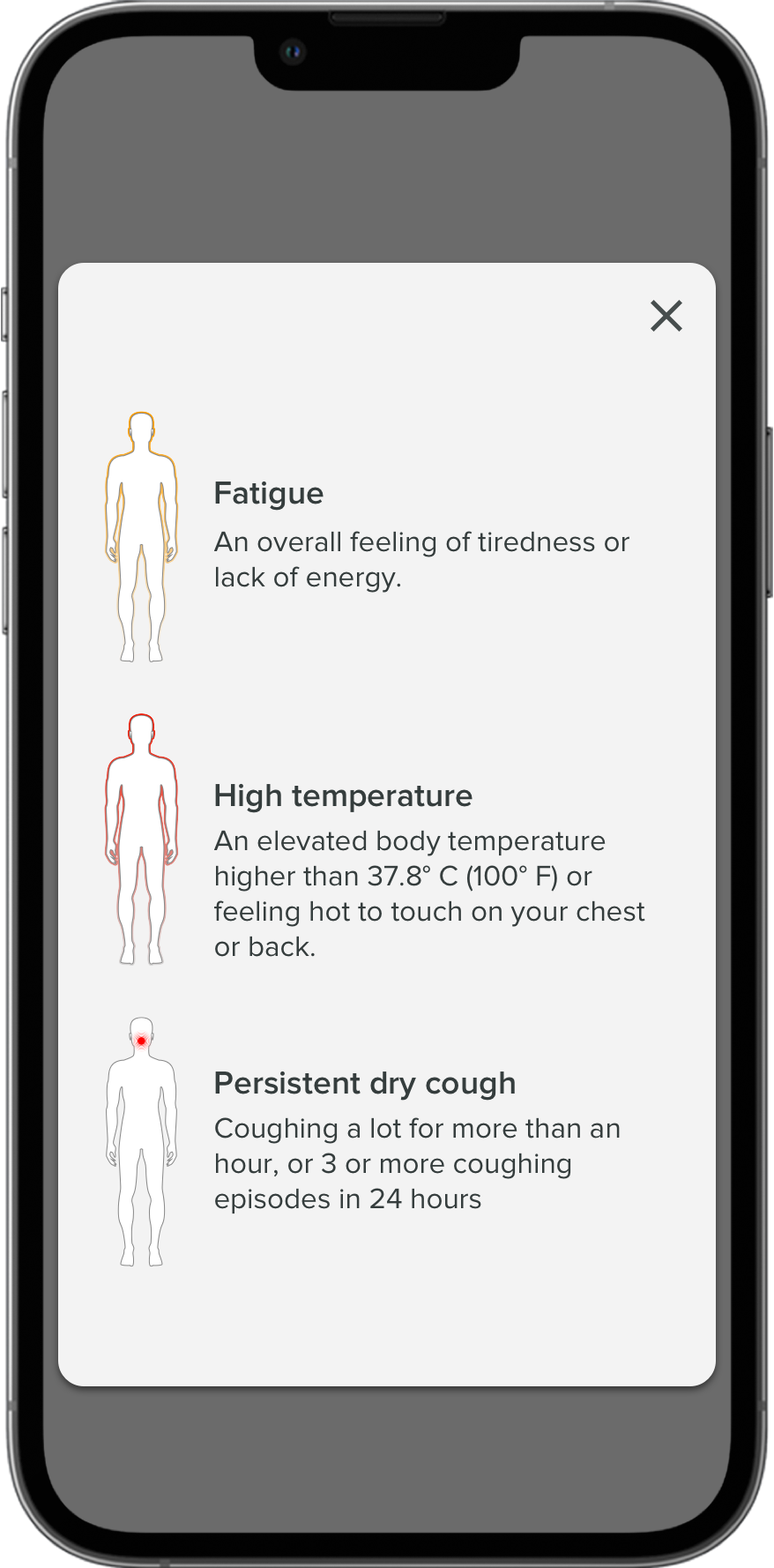 | 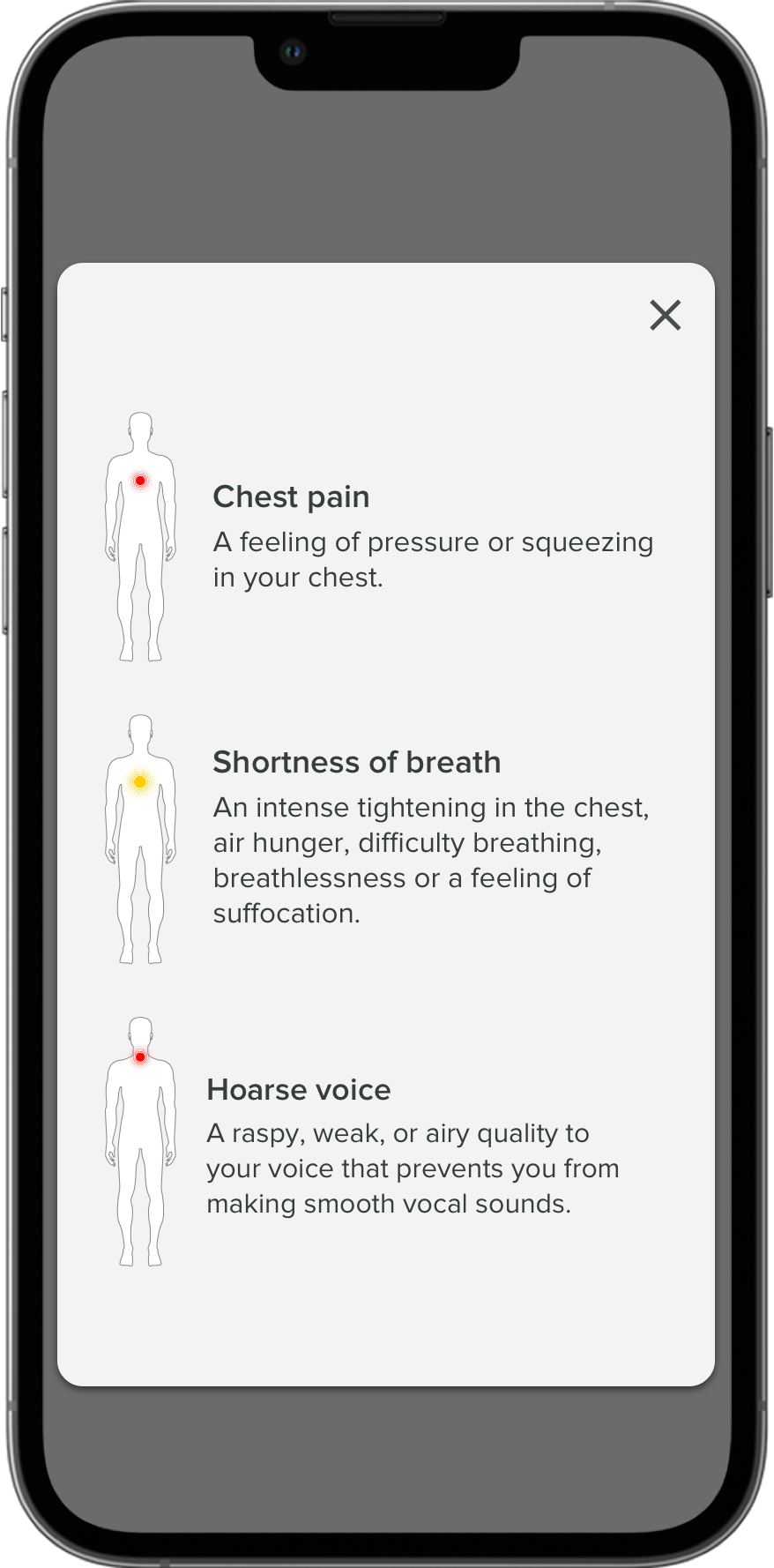 |
| Morning and evening routine management | |
| 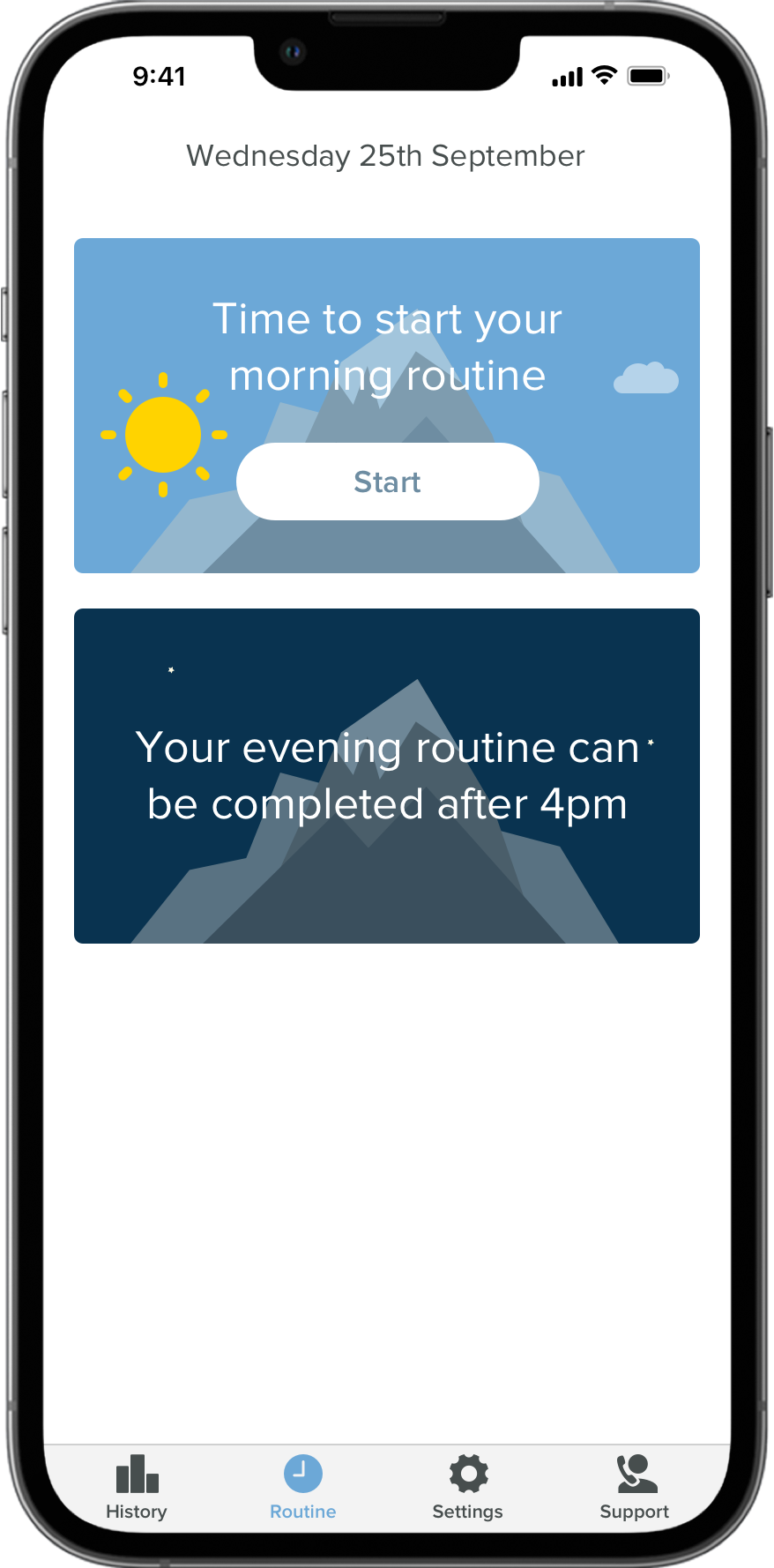 | 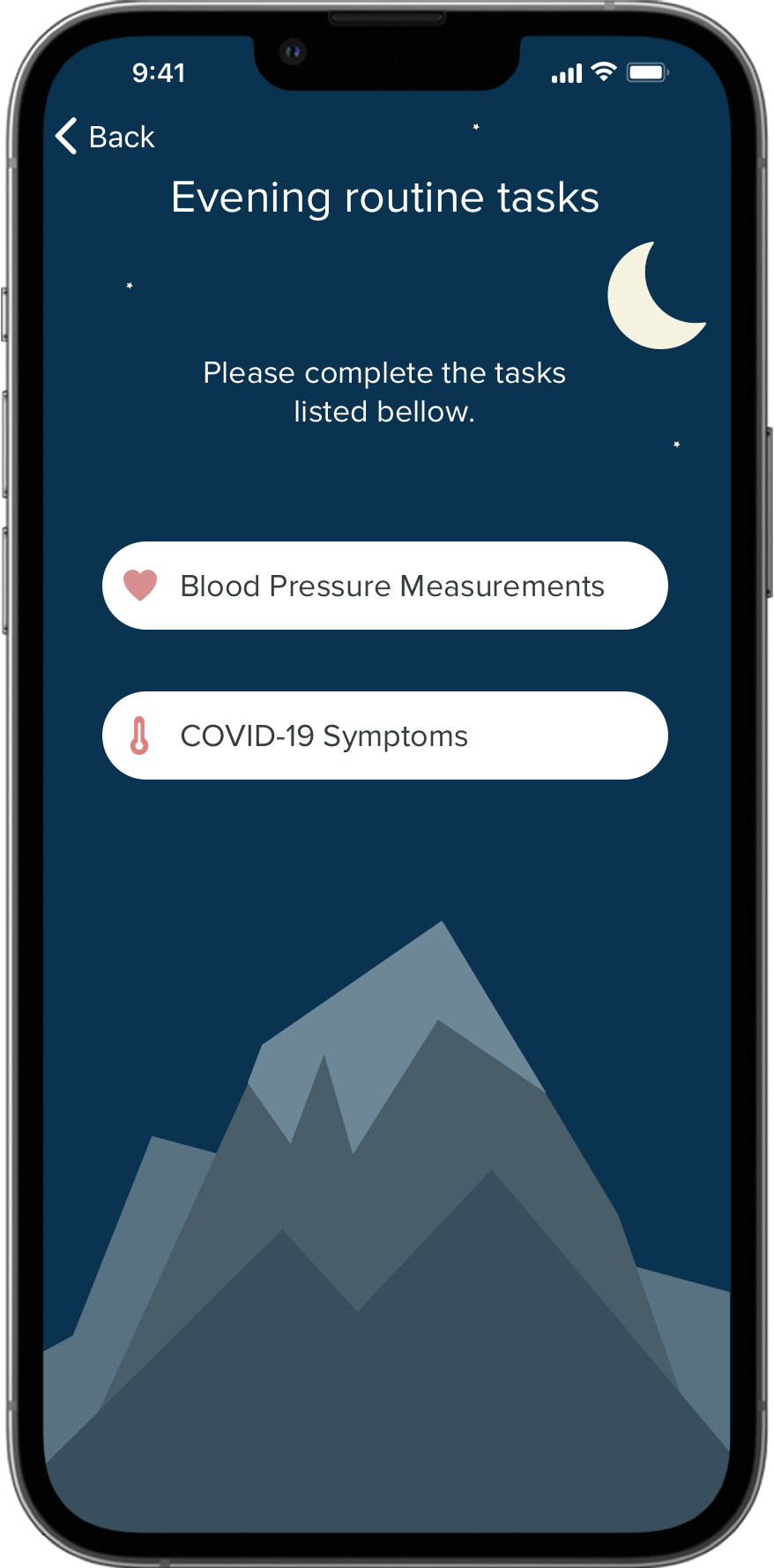 |

| Smart phone reminders | |
| --- | --- |
| 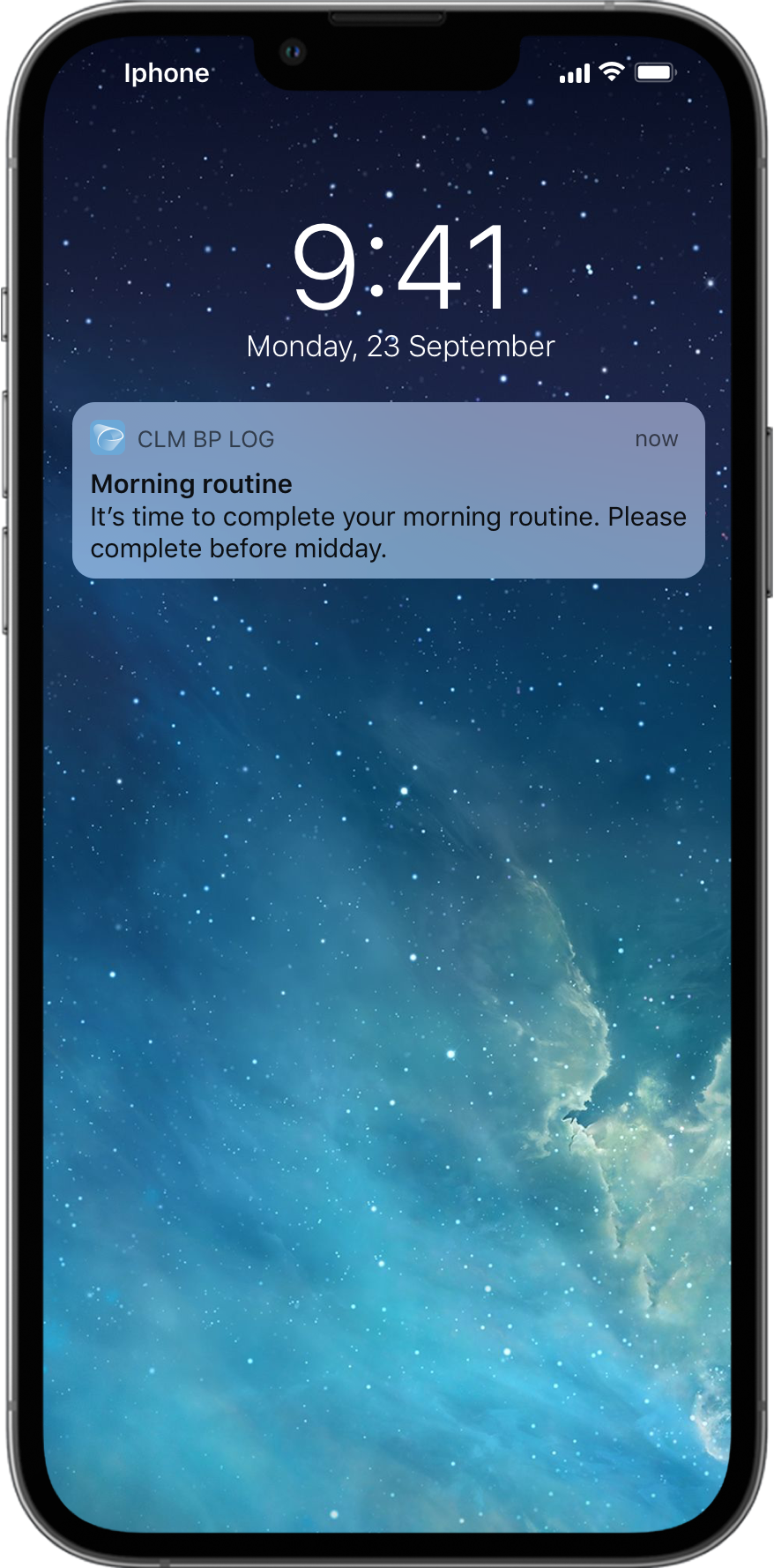 | 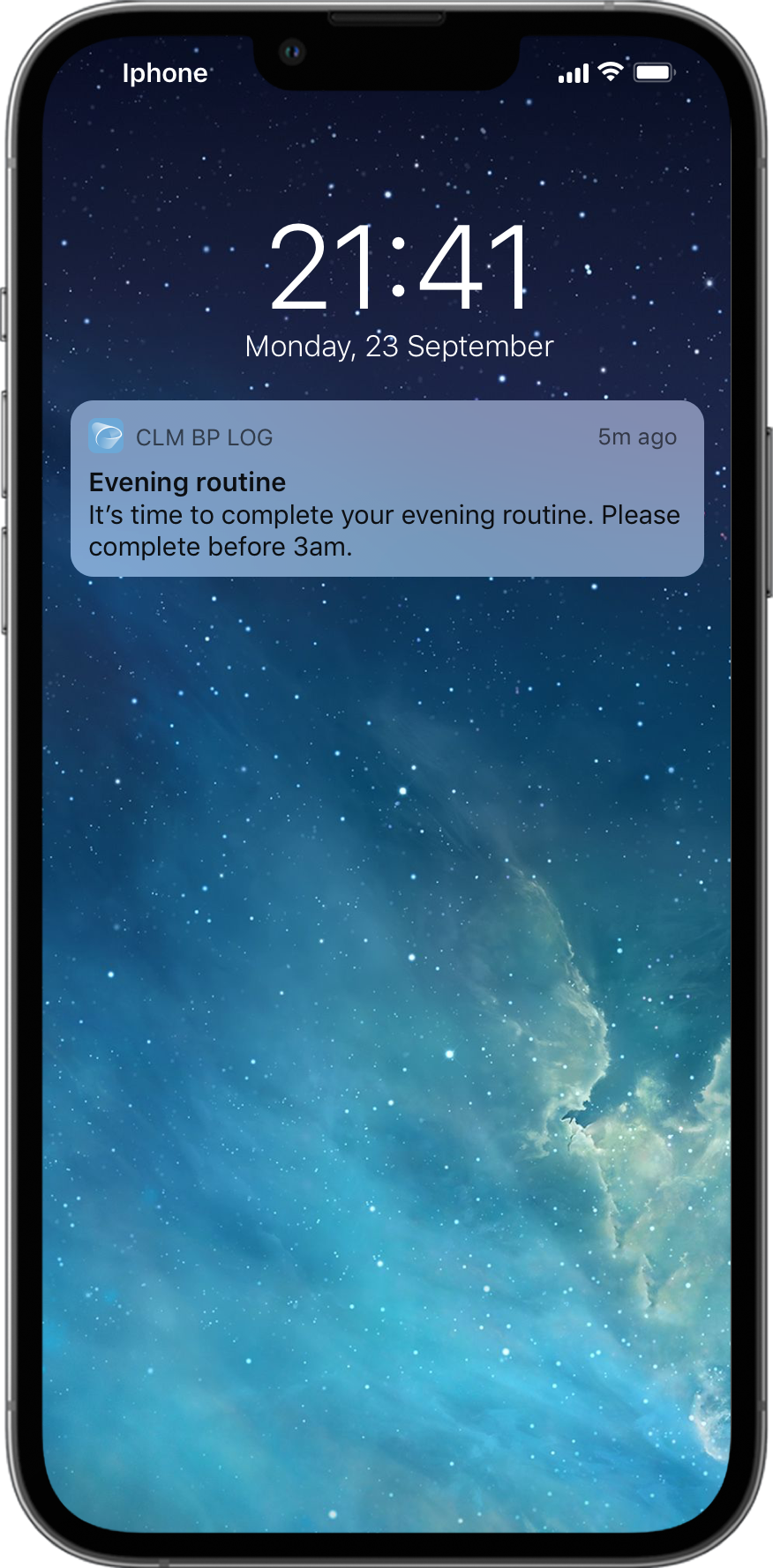 |
| Data history | |
| 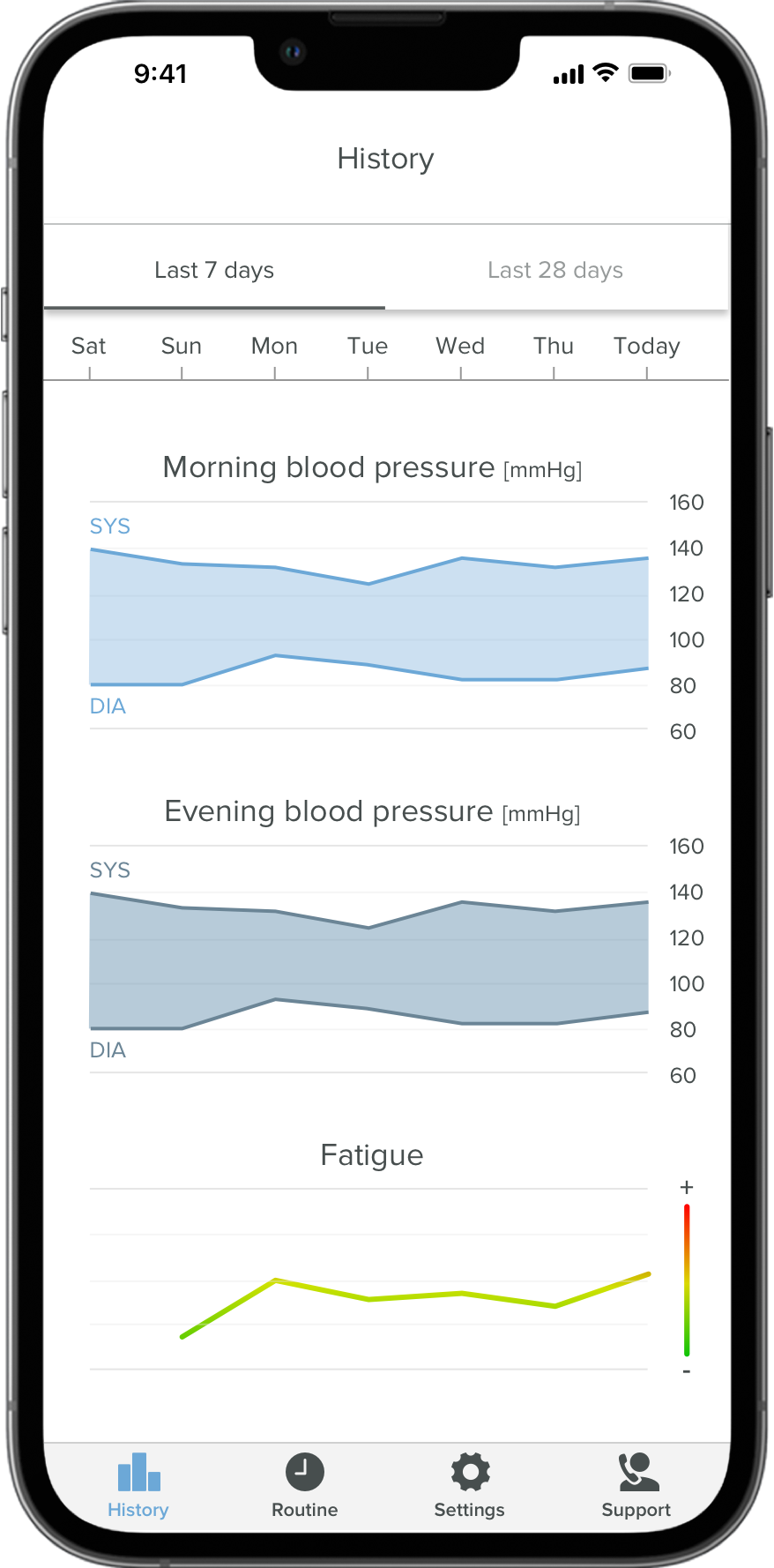 | 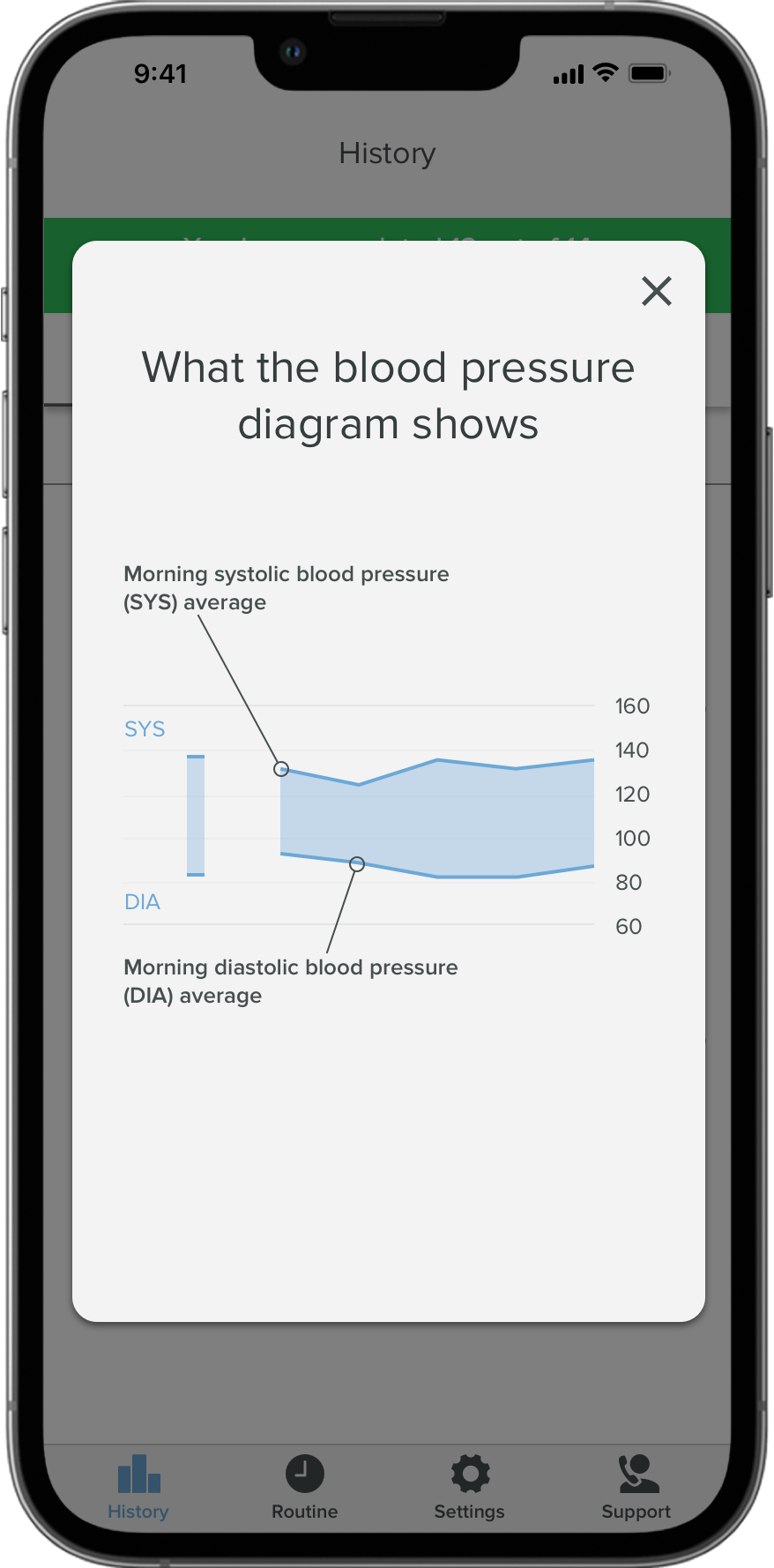 |
