# Supplementary figures and images for: Personalized Smartphone-Enabled Assessment of Blood Pressure and Its Treatment During the SARS-CoV-2 COVID-19 Pandemic in Patients From the CURE-19 Study: Longitudinal Observational Study
Source: JMIR Mhealth Uhealth. 2024 Dec 3;12:e53430. doi: 10.2196/53430 (PMC11653031; doi:10.2196/53430)

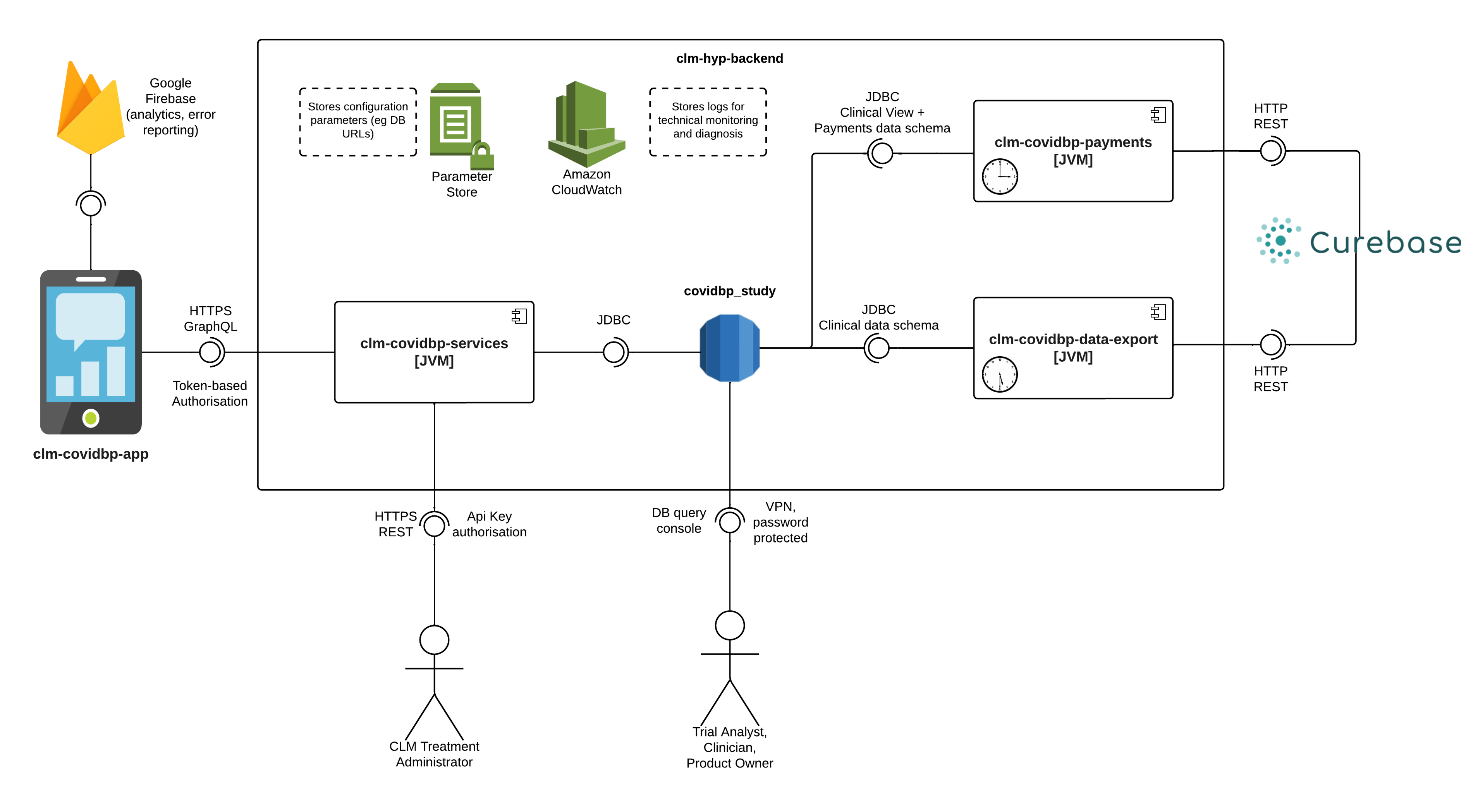

Supplement: Multimedia Appendix 2 [file mhealth_v12i1e53430_app2.png]

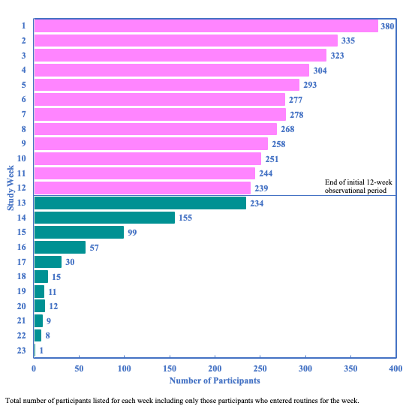

Supplement: Multimedia Appendix 3 [file mhealth_v12i1e53430_app3.png]
